# Distinct fine-scale variations in calcification control revealed by high-resolution 2D boron laser images in the cold-water coral *Lophelia pertusa*

**DOI:** 10.1126/sciadv.abj4172

**Published:** 2022-03-18

**Authors:** Jan Fietzke, Marlene Wall

**Affiliations:** 1 GEOMAR Helmholtz Center for Ocean Research Kiel, Wischhofstr. 1-3, 24148 Kiel, Germany.; 2 Alfred Wegener Institute, Helmholtz Centre for Polar and Marine Research (AWI), Am Handelshafen 12, 27570 Bremerhaven, Germany.

## Abstract

Coral calcification is a complex biologically controlled process of hard skeleton formation, and it is influenced by environmental conditions. The chemical composition of coral skeletons responds to calcification conditions and can be used to gain insights into both the control asserted by the organism and the environment. Boron and its isotopic composition have been of particular interest because of links to carbon chemistry and pH. In this study, we acquired high-resolution boron images (concentration and isotopes) in a skeleton sample of the azooxanthellate cold-water coral *Lophelia pertusa*. We observed high boron variability at a small spatial scale related to skeletal structure. This implies differences in calcification control during different stages of skeleton formation. Our data point to bicarbonate active transport as a critical pathway during early skeletal growth, and the variable activity rates explain the majority of the observed boron systematic.

## Teaser

Coral skeletal high-resolution boron systematics can shed new light on a still enigmatic control of coral calcification.

## INTRODUCTION

Many corals build an aragonite skeleton, a highly functional, mineralized structure that shelters its polyps, shapes ecosystems, and has persisted since the Ordovician (~450 million years). Organisms exert strong control on the calcification process, although conditions in the surrounding environment can also influence calcification. A comprehensive conceptual understanding of coral calcification is a long-standing scientific endeavor bringing together disciplines like biology, Earth science, chemistry, and more [e.g., (*1*–*4*)].

One prominent calcification model suggests that calcification occurs in an extracellular space [calcifying fluid or extracellular calcifying medium (ECM)], in which the chemistry can be modified by the living coral (*5*–*7*). This micrometer-sized fluid pocket is located between the skeleton and the calicoblastic cell layer of the coral tissue as constrained by electron microscopy (*8*–*10*). Seawater was shown to directly enter the ECM via paracellular passage (*11*, *12*), but the quantity of the so-called “seawater leakage” relative to material supplied to the ECM through transcellular transport is an active field of research. Typically, it is assumed that seawater enters the site of calcification, and the coral actively modifies the initial seawater-like chemical composition of the ECM (*13*, *14*). This conceptual model involves transport of calcium ions as well as different carbon species into the ECM, while protons are removed (*15*, *16*) and ultimately creates favorable conditions for precipitation of calcium carbonate. Such conditions are characterized by high aragonite saturation (Ω, the ratio of the ion product ([Ca^2+^] × [CO_3_^2−^]) in solution to the solubility product of aragonite) (*6*, *17*). The ion transport is achieved through active exchange of Ca^2+^ and protons via plasma membrane Ca–adenosine triphosphatase (ATPase) (PMCA) pumps hosted in calicoblastic cell layers of the coral tissue (*16*, *18*). While “ion pumping” supplies Ca^2+^ ions, it also elevates the fluid pH (“pH up-regulation”), shifting carbonate chemistry equilibria and increasing the concentration of carbonate ions within the ECM. At the same time, it removes protons produced as aragonite forms, which would lower the pH and negatively affect calcification (first approximation of net effect: Ca^2+^ + HCO^3−^ => CaCO_3_ + H^+^). Dissolved inorganic carbon (DIC) is thought to be supplied passively by diffusion of CO_2_ (predominantly metabolic) (*19*, *20*) and/or actively by bicarbonate anion transport (BAT) (*21*). Another prominent calcification concept calls for intracellular formation of amorphous calcium carbonate (ACC) as a precursor for calcification (*22*–*24*). Despite its differences, ACC formation also involves similar mechanisms of fluid modification like, e.g., PMCA and BAT (*25*), and an ECM between the tissue and skeleton (*25*).

Boron (B) is abundant in seawater and occurs at trace elemental concentrations in coral aragonite (*26*). Similarities between the boric and carbonic acid equilibrium have led to the development and application of B-based proxies for key seawater carbon parameters, i.e., pH, [CO_3_^2−^] or DIC, and the reconstruction of seawater pH from the B isotope (δ^11^B) composition measured in marine bio-carbonates (*3*, *26*–*29*). The basic concept of using δ^11^B to reconstruct ocean pH relies on the observation that B exists in seawater in two species: trigonal boric acid B(OH)_3_ and tetragonal borate ion B(OH)_4_^−^ (*30*). The relative abundance of the coexisting species is determined by the boric acid equilibrium: H_2_O + B(OH)_3_ H^+^ + B(OH)_4_^−^, which is inherently depending on pH. The second cornerstone of the δ^11^B pH proxy is the equilibrium fractionation of B isotopes in both coexisting species (*31*–*33*). The isotopic composition of total B present in seawater is constant throughout the modern ocean at 39.61 per mil (‰), and this represents the isotope signatures of both species, boric acid favoring the heavier and borate ion favoring the lighter isotopes (*34*). The degree of isotope fractionation between boric acid and borate ions has been predicted from theoretical chemistry and experimentally to α = 1.0272 (*32*). The final fundamental aspect of the B isotope pH proxy requires that borate is exclusively incorporated into the carbonate lattice substituting for carbonate ions (*26*). According to the latter assumption that B in the carbonate would represent the isotopic composition of seawater, the borate only links carbonate-bound δ^11^B directly to that of the borate ion in seawater and allows for reconstructing seawater pH. In addition, the pH-dependent abundance of borate in seawater is considered to modulate the B concentration in the carbonate linking the [B/Ca]_carbonate_ to [B(OH)_4_^−^/CO_3_^2−^]_seawater_ (*29*, *35*, *36*).

To calibrate the carbonate-bound δ^11^B proxy, we conducted experimental studies in a range of marine calcifying organisms including scleractinian corals (*37*–*39*) as well as field studies along pH gradients (*40*–*43*). These studies on δ^11^B and B/Ca revealed considerable complexities associated with direct pH reconstruction from carbonate-bound δ^11^B. Commonly, such deviations have been labeled “vital effects,” and they are ubiquitous in corals and other calcifying organisms (*37*, *44*, *45*). The degree of deviations is species specific, linked to the organism’s physiology (e.g., pH regulation) that modulates B isotope fractionation (*46*, *47*). Physiological studies advanced our understanding of calcification regulation (*4*, *6*, *48*) and provided important links to critical transporters and their influence on carbonate-bound δ^11^B (*16*, *21*, *49*). As a complementary approach, investigations of δ^11^B and B/Ca can provide deeper insights into coral physiology and its response to changing environmental parameters, specifically carbonate chemistry (*3*, *17*, *50*). This can also shed light on intraspecific differences and abilities to acclimate to future changes (*17*, *50*, *51*). Notably, while proxies often represent an integrated signal of weeks to months of growth, physiological measurements reflect shorter temporal scales, and both lines of research advanced the mechanistic understanding of coral calcification. Yet, it is desirable to study skeletal chemistry at spatial scales that allow insights into smaller-scale (micrometer) variability, moving us closer to the dimensions at which the process of calcification takes place (submicrometer) (*52*–*55*).

**Fig. 1.**
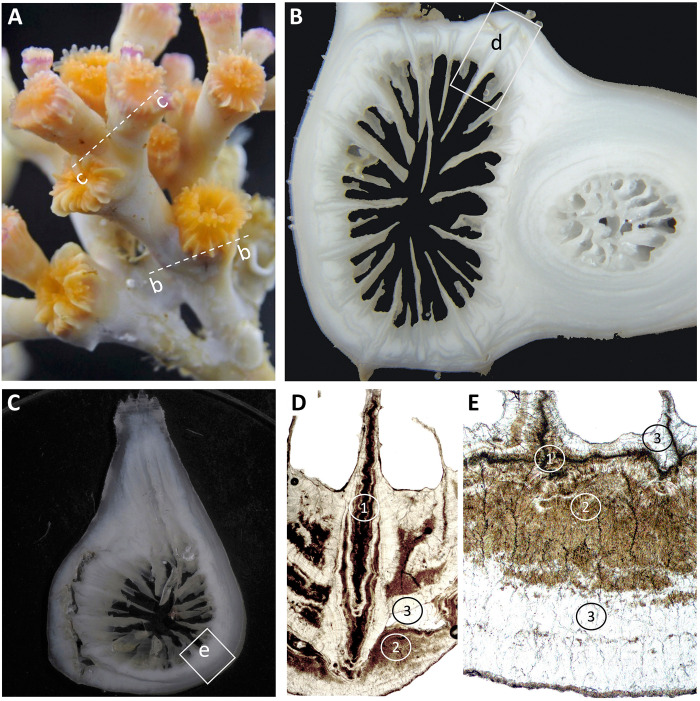
Example of *L. pertusa* specimen and different skeletal sections. (**A**) Living *L. pertusa* specimen (photo taken at the culturing facility at GEOMAR by J. Steffen) and indicated are the potential location of the different section in (B) and (C). (**B** and **C**) Light microscopic image of skeletal section that included two polyps. In (B), two polyps were cut transversal, one being recently formed while the other represent a rather basal part of the *Lophelia* colony with intense thickening, and (C) one polyp was cut longitudinally while the other was cut transversally. (**D** and **E**) Transverse polished section of septa and theca in transmission light microscopy showing dark brown zones of septal and septotheca early mineralization zones (EMZ) or rapid accretion deposits (1) and theca thickening deposits (2) that clearly separate from bright areas of fibrous aragonite or thickening deposits (3).

In this study, we used the skeleton of a cold-water coral *Lophelia pertusa* [now synonymous with *Desmophyllum pertusum*; (*56*)], a colony-forming azooxanthellate scleractinian coral (Fig. 1A) collected from its natural habitat off the Norwegian coast (see Materials and Methods). The *L. pertusa* skeleton shows characteristic skeletal features (Fig. 1, B to E) with distinct isotopic and elemental compositions (*45*, *57*, *58*) that may be related to calcification. This has led to the recommendation to avoid specific skeletal features and focus on fibrous aragonite in proxy applications (*59*). Here, we apply a laser ablation multicollector inductively coupled plasma mass spectrometer (LA-MC-ICP-MS) to simultaneously acquire high-resolution two-dimensional (2D) maps of δ^11^B and B concentration and link the distribution of structural features of the skeleton. The systematic relation between B and skeleton components suggests very different calcification control. Very low δ^11^B is characteristic to the skeletal region of rapid accretion. The observed low δ^11^B cannot be explained by common mechanisms like pH (and DIC) up-regulation of a seawater-like calcifying fluid, suggesting that the ECM is dominated by transport of seawater borate via bicarbonate transporters. In contrast, elevated δ^11^B in the theca wall is indicative of calcification from a pH up-regulated seawater-like ECM (with or without ACC as an intermediate precursor phase).

## RESULTS

**Fig. 2.**
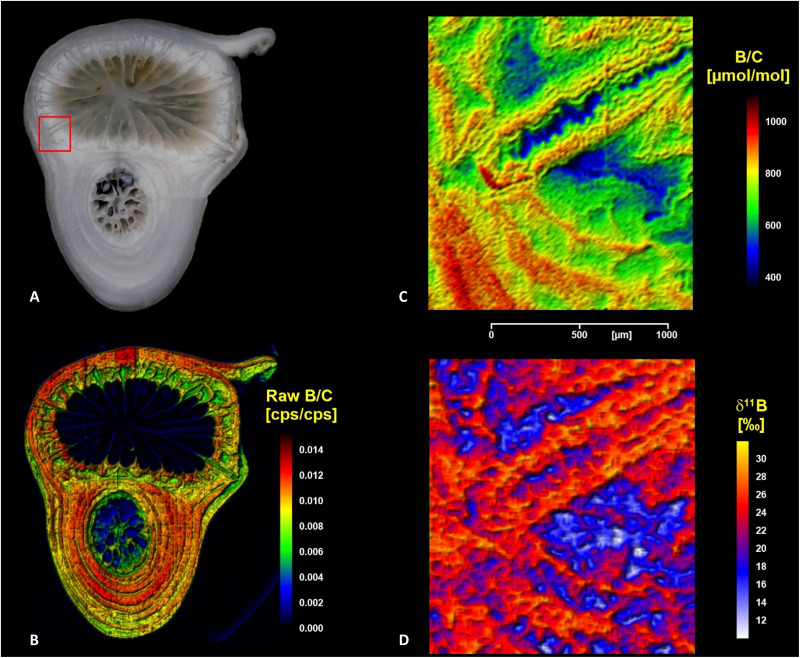
*L. pertusa* sample section boron laser images. (**A**) Optical image: Red rectangle indicating the area covered by high-resolution LA-MC-ICP-MS (C and D). (**B**) LA-MC-ICP-MS image of boron (^10^B + ^11^B) normalized to carbon (^12^C) ion intensity as a semiquantitative measure of boron concentration distribution [resolution: 100 μm (LA spot size) and 80 × 50 μm (data collection steps); image size: 17 × 15 mm^2^). (**C**) Boron concentration image based on calibrated LA-MC-ICP-MS boron (^10^B + ^11^B) normalized to carbon (^12^C) ion intensities. (**D**) Boron isotope (δ^11^B) image obtained by LA-MC-ICP-MS, measured against NIST-SRM610 standard glass and renormalized to NBS951. The area displayed in (C) and (D) [resolution: 20 μm (LA spot size) and 15 × 10 μm (data collection step size); image size: 1.7 × 1.5 mm^2^) is located within the skeletal part marked by the red rectangle in (A).

We obtained fully quantitative high-resolution 2D maps of both B concentration and δ^11^B at per mil–level precision (simultaneously measured) for aragonite skeleton of the cold-water coral *L. pertusa* at high spatial resolution [individual pixel size: 15 × 10 μm^2^; precision for individual pixel: 1.5 to 2‰ (2 SE); image size: ~17,000 pixels]. Analyses comprised correlative geochemical imaging using electron beam [electron microprobe (EMP); scan resolutions of 20 and 5 μm] and laser-based methods (LA-MC-ICP-MS; laser spot sizes of 100 and 20 μm for δ^11^B and B concentrations, respectively) (Fig. 2) (for more details, see Material and Methods and the Supplementary Materials).

The semiquantitative B/C overview map (Fig. 2B) has been used to evaluate the broader picture of B distribution, compare it to the optical features (Fig. 2A), and identify a particular area of interest for the consecutive high-resolution analysis (Fig. 2, C and D). The B/C distribution (indicator of B concentration) clearly resembles features in the optical image (Fig. 2A compared with Fig. 2B). In particular, high B/C values are associated with more translucent (darker gray in Fig. 2A) parts of the skeletal section (e.g., concentric growth layers in the lower sample part). Low B/C values can be found in optically opaque sample areas (whitish in Fig. 2A; e.g., centers of septa and neighboring theca in the upper sample part). For the high-resolution analysis, the focus was on an area that displayed large small-scale B/C variability associated with particular structural features of the skeleton, i.e., a septum and its neighboring theca wall (marked in Fig. 2A by a red rectangle, resembling skeletal features visualized with transmission light microscopy; Fig. 1C).

The high-resolution laser B images [approximately 17,000 pixels at 15 × 10–μm data collection resolution; Fig. 2C (B/C) and Fig. 2D (δ^11^B)] display a large degree of fine-scale (tens of micrometers) variability in B, covering a large total range in both concentration and δ^11^B [B concentration: 420 to 970 μmol/mol; δ^11^B: 8.5 to 32‰]. To a minor degree, analytical uncertainty [1.5 to 2‰ (2 SD) depending on local boron concentration] contributes to this measured variability in boron isotope ratios. At this high spatial resolution, we were able to clearly resolve fine-scale features of coral skeleton and link these features to B systematics. Focusing on areas most distinct with respect to B, we observe the following: Low B concentrations accompanied with low δ^11^B dominate the very center of the septum as well as the center of the neighboring theca wall (data cluster “LL-B: low B concentration and low δ^11^B”). For this data cluster, 90% of the data fall within a B concentration range of 540 to 680 μmol/mol and a δ^11^B range of 12 to 19‰. Notably, B concentrations gradually trend up along the apparent direction of growth outward and inward from the center of the septotheca, while δ^11^B maintains the low signatures. We provide an interpretation of that transition area in text S8. The centers of the septum and septotheca are relatively enriched in Mg, while both S and Sr are relatively depleted (see text S2 and fig. S2).

In contrast, high B concentrations with high δ^11^B (“HH-B: high B concentration and high δ^11^B”; as well as low Mg; see text S2 and fig. S2) can be found in growth layers thickening the septum (tens of micrometers thick) and in the outer layers of the theca wall (hundreds of micrometers thick). This HH-B data cluster ranges from 810 to 970 μmol/mol in B concentration and δ^11^B from 24 to 29 ‰, respectively.

## DISCUSSION

The unique application of LA-MC-ICP-MS as an imaging tool provides the most detailed and comprehensive insights into the fine-scale variability of B concentration and δ^11^B within a coral skeleton reported to date. The total δ^11^B range observed in the high-resolution image [δ^11^B: 8.5 to 32‰; see Fig. 2D] of *L. pertusa* in this study exceeds the ~10‰ range reported for this particular species (*43*, *45*, *57*, *60*). Past *L. pertusa* δ^11^B studies used ion probes and discrete point measurement data. While this method can produce accurate information, discrete point measurements can easily miss small-scale variability and thus associated variation caused by calcification control. Our approach allows for a direct link of skeletal features [e.g., banding patterns observed within early mineralization zone (EMZ)] with δ^11^B and thus micrometer-scale observations that corroborate variability in organisms caused by calcification.

Earlier studies proposed several explanations for the observed large δ^11^B range in *L. pertusa*. Blamart *et al.* (*45*) claimed that skeletal δ^11^B in *L. pertusa* is not related to seawater pH and pointed out that the observed vital effect with low δ^11^B in the EMZ cannot be explained by a simple ECM pH up-regulation model. They concluded that different physiological control mechanisms need to be active during two distinct phases of skeleton formation (EMZ versus fibrous aragonite), as also supported by O and C data. Others focused on the pH up-regulation capacity of *L. pertusa* in the theca wall and found that corals regulate the pH in the ECM to comparable levels regardless of the external water pH (*56*). In contrast to Blamart *et al.* (*45*), Jurikova *et al.* (*60*) interpreted the δ^11^B offsets between EMZ and the theca wall as indication of two pH endmembers, both reflecting the pH conditions in the ECM. They proposed that the skeleton within the EMZ forms from an ECM with elevated DIC but seawater-like pH (hence low in δ^11^B) and that the fibers of aragonite are deposited from a pH up-regulated within the ECM (i.e., high δ^11^B).

**Fig. 3.**
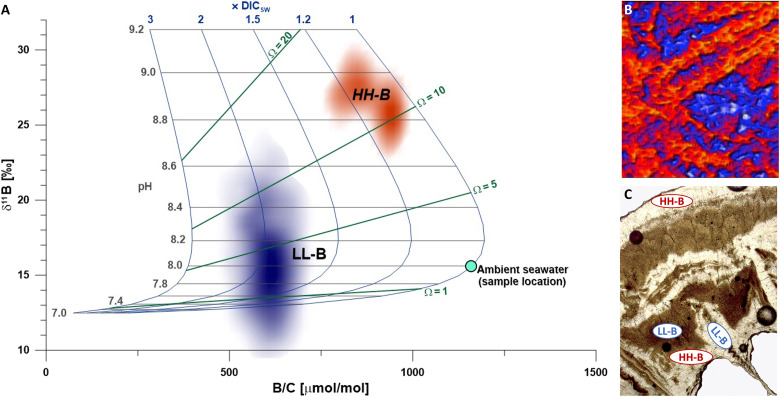
Modeled relationship between ECM carbonate chemistry and aragonite boron systematics and its links to structural components. (**A**) Combined boron isotope and concentration results from specific sample areas of *L. pertusa* high-resolution laser images (HH-B: high B concentration/high δ^11^B in outer theca wall and layers covering the septum; LL-B: low B concentration/low δ^11^B found in innermost parts of the septum and theca wall). The underlying boron carbonate chemistry grid is based on calculation of B isotope ratios and concentration values of aragonite formed from an ECM derived from seawater and the modification of the carbonate system parameters pH and DIC. Lines of specific aragonite saturation (Ω) are displayed to contextualize the results of both B data groups. Ω calculation uses seawater Ca concentration of 10.4 mM. To account for differing Ca concentration, the displayed Ω needs to be multiplied by [Ca]_CF/ECM_/[Ca]_seawater_. (**B**) High-resolution boron isotope image for spatial identification of HH-B (red) and LL-B (blue) areas. (**C**) Transmission light microscopic image for the identification of typical structural components associated with HH-B, resp. LL-B composition (note: a separate section of the sample needed to be prepared as thin section for this image).

Established concepts often target aragonite supersaturation conditions that require up-regulation of pH and DIC (*3*, *29*). Because B proxies are expected to reflect carbonate chemistry conditions, both HH-B and LL-B data can be translated into pH and DIC and thus estimate Ω within the ECM (Fig. 3). This boron systematic displayed in Fig. 3 is based on initial seawater conditions at the site of sample collection (pH_total scale_ of 8.0, DIC concentration of 2150 μmol/kg) and considers only the modification of pH and DIC (see text S5 for more details). In this respect, the HH-B cluster is consistent with the established concept of aragonite supersaturation by pH and DIC up-regulation and found within the theca wall and the thickening deposits of the septum. In this case, the corresponding carbonate chemistry conditions within the ECM would have, on average, increased by 0.87 ± 0.16 pH units relative to seawater and are accompanied by only a small increase in DIC (~5 to 25%). This DIC influx into the ECM is likely supplied by respiratory CO_2_ diffusing across the cell membrane into the ECM driven by the pH/CO_2_ gradient between calicoblastic cells and ECM (*19*, *20*). The resulting Ω (~8 to 14) represents a four- to eightfold increase relative to the ambient seawater Ω for aragonite of 1.8. These values are in the range of expected saturation state conditions from inorganic aragonite precipitation experiments that produce crystal structures similar to coral fibers as seen in the thickening deposits (*60*).

The LL-B data cluster structurally corresponds to EMZ both in the center of septa and the theca wall (Fig. 3C and texts S4 and S6). In contrast to the HH-B, its B composition cannot be explained by pH/DIC up-regulation. The respective ECM under that scenario would have to contain almost twice the DIC concentration of seawater (>4000 μmol kg^−1^) at a pH equaling that of the surrounding seawater. While this has been proposed by Jurikova *et al.* (*60*), Blamart *et al.* (*45*) argue against this based on the differences in O and C isotopes observed in EMZ and fibrous aragonite. Specifically, δ^18^O indicates an elevated pH during skeletal formation of the EMZ and further supports that conventional B systematics formation is not applicable for this region. (Both C and O isotopes have the potential to provide unique additional insights and address underlying assumptions. Some insights and issues related to these isotopes will be discussed in text S9.) Moreover, the respective ECM conditions would represent only a small increase in aragonite saturation state Ω to 2 to 3, arguably low and in stark contrast to hypothesized supersaturated EMZ skeletal regions (*61*). Similarly, observations from inorganic precipitation experiments underline that supersaturation (Ω > 20) fosters crystal nucleation and the formation of EMZ-like granular crystals and supports the idea of supersaturation (*62*). These estimates for Ω (4- to 8-fold increase in HH-B and 1- to 1.5-fold increase in LL-B skeleton) are at the lower range of values, because they do not account for any increase in ECM’s calcium concentration resulting from the Ca^2+^/H^+^ pumping or transport of calcium by Ca channels (*63*). Calcium concentrations in the ECM of tropical corals have been measured and are elevated compared with seawater [11 to 15 mM; (*6*, *48*)], which may also be the case for cold-water corals like *L. pertusa*. Thus, calcium could be elevated to reach similar or even higher Ω within the LL-B at the B-derived DIC and pH levels. However, calcium would need to be a lot higher, approximately three to five times that of seawater (~30 to 50 mmol/kg). To reach such high calcium concentration, this would require large amounts of protons to be pumped out of the ECM, resulting in a major increase in ECM pH and consequently high δ^11^B. That is clearly not consistent with the LL-B data cluster. In this context, it needs to be noted that Ca transporters that do not affect pH [e.g., voltage-gated calcium channels without H^+^ cotransport (*62*) or Na^+^/K^+^-ATPase calcium cotransport; (*49*)] may operate to provide calcium to the ECM. Still, we consider such high calcium concentrations as not realistic, and they have not been measured to date. In addition, a notable portion of the LL-B data plots below Ω = 1, conditions under which aragonite would not precipitate. This light δ^11^B is practically impossible to be explained by a simple pH-driven boric acid equilibrium within a seawater-like (with respect to B) ECM, because it would imply unrealistically low pH (<<8).

The regions of the LL-B, the EMZ, are considered to be rapid accretion deposits, suggesting faster aragonite growth rates (*64*, *65*), which would require higher saturation conditions than currently discerned from simple pH-DIC up-regulation [note that we used the term EMZ in this study because it is more commonly applied in coral boron work, but it was suggested that rapid accretion deposits are a more appropriate term for this skeletal region; (*65*)]. Thus far, no clear explanation has been provided that can resolve the observed B variability among different *L. pertusa* skeletal parts and identify the underlying physiological mechanisms. Together, this underscores the need for a completely different view on the processes controlling B at the site of calcification. In line with previous suggestions on compartmentalized calcification (*66*, *67*), we propose two different physiological control mechanisms that modulate the B systematics during calcification and can explain the contrasting differences. The proposed pathways combine variations in pH and DIC transport that differ significantly during calcification of different skeletal structures.

The discrepancy between the need for high Ω to support nucleation and rapid growth and the calculated low aragonite saturation conditions (Ω 2 to 3) for the EMZ requires a novel explanation. The very light δ^11^B values of the EMZ suggest precipitation from a source with δ^11^B lighter than seawater. This precludes a scenario in which the ECM initially starts with a seawater B composition, evolving via the precipitation process, without additional influx of B. The preferential removal of lighter B into the forming aragonite would over time gradually shift the remaining B in the ECM toward heavier composition (i.e., Rayleigh fractionation). Similarly, seawater leakage as a dominant transport mechanism of B into the ECM can be excluded. The amount of B incorporated into aragonite is very small even at high precipitation rates. Therefore, removal of B from the ECM would be very slow, and B in the ECM would remain in equilibrium with seawater B. Actually, the mean B composition of the LL-B group closely resembles that of seawater borate at the ambient seawater pH of 8.0. Thus, we could assume that seawater borate is the dominant B source to the ECM. Seawater borate has an isotopic signature of 15.6‰ and, if influx and outflux are in balance, imprints this signature into the newly precipitated carbonate. In such a scenario, it is important to understand how seawater borate may be transported.

A conceivable candidate for the transport of seawater borate into the ECM is BAT, like members of the solute carrier 4 family localized in coral calicoblastic cells (*18*, *21*, *49*)). Those transporters allow for the transport of bicarbonate ions into the ECM, supplying DIC, and they have been described as putative B (more precisely borate) cotransporters to a limited extent (*21*, *68*). Because recent studies identified them as critical for calcification, they have become more commonly integrated into calcification models (*3*, *21*, *69*) and suggested to play a key role in calcification similar or even superior to PMCA (*49*). A gene expression study in the tropical corals *Acropora palmata* and *Acropora cervicornis* found that multiple PMCA transcripts in the fast-growing tip were not expressed stronger compared with the slow-growing basal part, while some BATs were higher expressed (*70*). Such findings provide a first hint on the importance of BATs in fast calcification. Despite the fact that localized differences in transport mechanisms have been suggested before (*54*, *67*), thus far, no model considers their relative importance and their potential for driving localized differences in growth and isotopic signature. The efficiency of borate cotransport by BAT does not need to be particularly high. It only needs to be in the order of the ratio of B to C measured in the aragonite (~0.5 to 1 × 10^−4^) to balance the influx and outflux (I=O) of the respective ions for the ECM. Active BAT has a direct additional benefit for the calcification, as it also contributes and supports pH regulation (*21*, *49*). For instance, 1 mol of bicarbonate as DIC source used for calcification releases just 1 mol of protons during conversion into carbonate ions in contrast to 2 mol released from converting 1 mol of CO_2_ (e.g., from respiration and diffusion into the ECM, fostered by the catalytic activity of carbonic anhydrase). Therefore, when BATs are activated, the same Ca^2+^/2H^+^ pumping rate results in a more effective pH up-regulation and ultimately stronger increase in the aragonite Ω. This would result in an increased precipitation rate as has been proposed for the EMZ. To maintain enhanced precipitation, the DIC supply needs to keep pace with the high demand. The function of BAT is precisely providing additional DIC to the ECM. This process is consistent with the suggestion that seawater leakage is likely not a dominant source of B within the EMZ. Two scenarios are possible: (i) entire shutdown and no exchange with seawater during calcification or (ii) reduced leakage that is overwhelmed by transcellular transport. We consider (ii) to be more realistic because coral tissue was found to be permeable for large molecules (calcein), suggesting that seawater can directly enter the ECM (*11*, *12*). It needs to be noted, however, that epithelial resistance may be high, a fact that significantly restricts the ion fluxes from seawater to the ECM (*12*, *71*). We note, however, that the epithelial resistance experiments have been carried out on symbiont-bearing tropical corals and did not investigate differences between EMZ or fibrous aragonite precipitation. The direct transferability of these results to EMZ formation in *L. pertusa* warrants further in-depth and targeted physiological investigation.

Several studies modeled the chemical composition of the ECM, primarily not only focusing on carbonate chemistry but, to some extent, also including B systematic. Chen *et al.* (*72*) examined the role of carbonic anhydrase in the reaction kinetics within the ECM, budgeting the fluxes of seawater leakage, PMCA-controlled Ca^2+^/H^+^ pumping, diffusion of metabolic CO_2_, the catalytic transformation of aqueous CO_2_ into HCO_3_^−^, and the ultimate output flux by aragonite precipitation. Their preferred model with fast carbonic anhydrase kinetics matches the ECM conditions derived for theca and fibrous skeletal parts in this study. However, their model cannot produce results that match the apparent ECM chemistry parameters for EMZ skeletal parts (see Fig. 3). Likewise, the model calculations of Guo (*69*) agree with our findings for fibrous aragonite but do not explain the B data we observe in EMZs. Guo (*69*) mentions BAT as one of the key processes involved in calcification but, to our understanding, did not explicitly account for it in his model. Gagnon *et al.* (*73*) combine ECM modeling with B data from a set of coral culturing experiments. Their model considers transmembrane diffusion of boric acid into the ECM as a key process to shift the ECM B isotope composition toward lighter values, masking its pH dependency. Skeletal B systematic in their model is driven by a balance between B influx via seawater leakage and boric acid diffusion. While their model allows for δ^11^B values as low as 15.5‰, it cannot explain the EMZ δ^11^B as low as 8.5‰ we observe. We take this as indication that boric acid diffusion is not the main driver for the B systematic in EMZs.

**Fig. 4.**
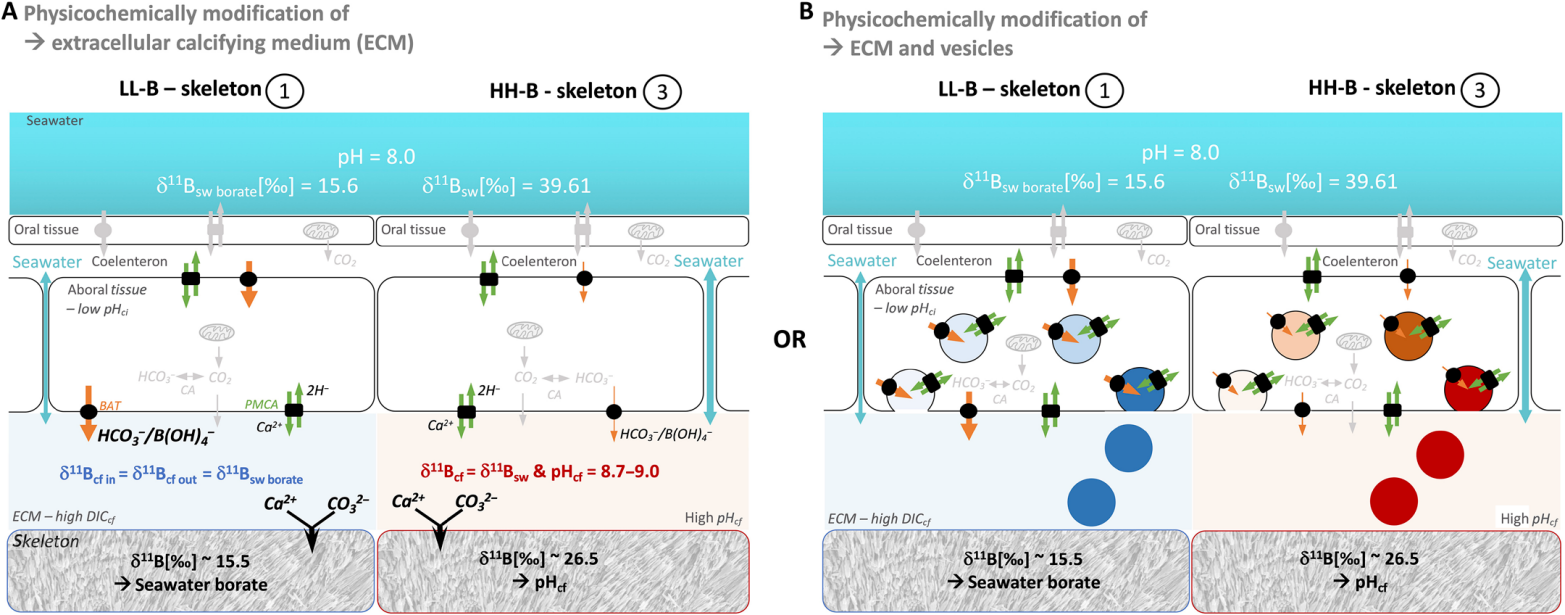
Proposed concepts for the boron transport from seawater to the coral skeleton. We provide potential pathways that lead to the formation of coral skeletal structures with distinct boron (B) signature (light B isotope and low B concentration—LL-B; heavy B isotopic and high B concentration—HH-B) using common calcification models (**A**) physicochemical modification of ECM only or (**B**) modification of ECM and cell vesicles (*24*). (A) The transport of boron into the ECM is dominated by either seawater leakage via a paracellular pathway (right part, HH-B) or active BAT that cotransports borate [B(OH)4^−^] via a transcellular pathway (left part, LL-B). From the ECM, the skeletal aragonite is precipitated extracellularly. (B) Boron transport comparable to (A) but including vacuolization from the ECM and intracellular precipitation of an ACC precursor phase. All transporters in color (orange; green indicating BAT and PMCA) are directly relevant for our calcification modes and effect boron systematics. Other relevant transporters are indicated in gray (e.g., CA—carbonic anhydrases or CO_2_ diffusion). Numbers next to LL-B or HH-B skeleton (1, 3) link the skeletal part presented in Fig. 1.

We propose a calcification model that involves B transport pathways and is consistent with the B observed in the skeletal aragonite of *L. pertusa*. We find the simultaneous formation of different skeletal structures at different locations throughout the corals’ skeletal tissue (*64*) and propose that they form via mechanisms that predominantly use different transporters. Overall, this model supports the concept of a compartmentalized calicoblastic cell layer (*66*, *67*), and like the compartmentalized calicoblastic cell-layer model, we cannot tell whether different cell types exist or similar cells are responsible for the distinct formation by operating in different modes and/or modulating the activities of various transporters. The formation of EMZ skeleton (Fig. 4A, left) requires a high BAT activity that supplies DIC to the ECM in addition to the diffusion of respiratory CO_2_ from the cells of the aboral tissue. Borate cotransported by BAT dominates the δ^11^B signature of the input flux into the ECM in our model. Calcium supply and proton removal are facilitated by PMCA exchanging Ca^2+^ for 2H^+^, elevating the pH and, consequently, the aragonite saturation state Ω. Because both BAT and Ca-ATPase pumping simultaneously provide efficient pH and DIC up-regulation (and also increase the gradient in pH and CO_2_ and strengthen the diffusive CO_2_ influx), high Ω and consequentially high precipitation rates are achieved. Maintaining this calcification mode may result in a higher demand of energy for the extra activity of BAT. The observed colocalization of Na^+^/K^+^-ATPase with BAT (*49*) as well as elevated expression of Na^+^/K^+^-ATPase at fast-growing skeletal regions (*70*) requires energy can be responsible for energizing BAT activity (*49*) and, hence, a conceivable scenario for the calcification process of *L. pertusa*. This proposed calcification mode results in the precipitation of new skeletal aragonite from the ECM carrying the light δ^11^B signature of its seawater borate source. B concentration of that aragonite is thus defined by the rate of borate cotransport by BAT [or to the extent BAT discriminates against H(BO)_4_^−^ while transporting HCO_3_^−^].

Large parts of the theca wall, the skeletal fibers, or thickening deposits follow conventionally described control mechanisms dominated by pH up-regulation (*41*, *46*). Here, BAT activity (if happening) is overwhelmed by seawater leakage that needs to be the dominating source of B supply to the ECM (Fig. 4A, right). pH up-regulation likely via PMCA results in an elevated aragonite saturation state Ω. The absence of BAT activity and thus restricted DIC supply would result in lower Ω compared with the previous scenario and consequently lower precipitation rates. This calcification mode is more likely energy saving and used by the coral for most of its skeleton thickening deposition. The low consumption of B during aragonite precipitation and the “openness” of the ECM to seawater keep the ECM equilibrated to seawater with respect to B. During this mode, B in the aragonite carries the isotopic signature of borate from a seawater-like ECM as modified by the ECM’s pH. Previous studies described EMZ-like zones within the theca wall with low δ^11^B (*45*, *57*), and these growth rings are also seen in the B concentration maps (Fig. 2B and fig. S5). To date, we do not know for certain whether this represents a certain periodicity, e.g., yearly growth marks, but this could reflect shifts in calcification control and, for instance, the initiation of a new growth cycle.

So far, we focused on the often-used calcification model that regards “physicochemical” dominance to explain proxy modulations, in contrast to an ACC “biologically controlled” calcification model. The potential role of an ACC precursor in coral calcification has been discussed for quite some time and has recently gained new attention. High-resolution methods enable the mapping of ACC at the growing rim of diverse corals (*25*, *74*), and cell studies provide further evidence of vesicle activity in calicoblastic cells (*75*). Thus, we explored whether changes in B composition comply with the ACC calcification model (Fig. 4B). As suggested in a recent model, the same transporters that enrich the ECM also control the vesicle conditions (*25*), hence resembling the controlling mechanisms described before. The main difference is the actual fluid volume that is manipulated. In the ACC scenario, it is primarily the vacuoles with possible additional active modification of the ECM. Again, BAT accounts for the main difference with respect to the resulting B composition of the skeleton. Yet, vacuolization from the seawater and transportation toward the skeletal side (*74*) seems unlikely, because the amount of B carried by seawater vacuoles is large and would be hard to overprint by the BAT cotransport of borate. An updated model already proposed an ECM route (*25*) together with a dual mechanism of skeleton growth by ACC attachment and ion-by-ion growth. In particular, the latter underscores the importance of biological control on the physicochemical conditions within the ECM and supports the notation that there is no calcification in corals without biological control. Note that ACC models also emphasize the role of organics in calcification and stabilization of ACC. Although organics are part of coral skeletons, we consider them negligible in terms of skeletal boron signature in this case (see text S7).

While we cannot exclude either of the proposed models here (Fig. 4, A and B), it may also be possible that the relative contribution of ACC and ion-by-ion growth varies. It is plausible that within the EMZ, ACC-driven high vesicle activity dominates the calcification process, compared to areas of skeleton thickening with predominant ion-by-ion growth. Such scenarios may differ within and between different coral species and should be a research focus of future studies.

In summary, our proposed concepts of differing ECM control for different skeletal regions are one conceivable option consistent with both analytical findings and established model concepts of coral calcification. This process likely operates in other corals and calcifying organisms and could be tested. High-resolution imaging of coupled B concentration and δ^11^B allows for linking details of chemical composition to skeletal structural features. It provides complex datasets to test models of calcification processes and can provide crucial information regarding the variable physiological control (transport mechanisms) an organism applies on the ECM and ultimately on calcification. Because B isotopes are also used by geochemists as a proxy for seawater pH, our findings offer fascinating new opportunities. Our approach may guide us to look for parts of the skeletons in other corals/calcifying organisms, which directly incorporate the δ^11^B of the surrounding seawater, without additional fractionation allowing for the unbiased reconstruction of seawater pH and no assumption on the organism-specific degree of pH up-regulation. Last, the concept proposed can result in targeted geochemical studies to assess the effect of this calcification process on isotopes and concentration of other elements.

## MATERIALS AND METHODS

In this study, we have used an *L. pertusa* specimen collected in September 2011 during *Poseidon* cruise POS420 from a living colony at outer Trondheim Fjord near the Norwegian island Nord-Leksa (63°36.4′N, 09°22.7′E) from 145- to 220-m water depth (*76*). The seawater at this location has a temperature range of 7.6° to 7.9°C, a salinity of ~35, a pH_total scale_ of 8.0, a total alkalinity of 2300 μmol/kg, and a DIC concentration of 2150 μmol/kg.

The skeletal sample has been embedded in epoxy resin sectioned using a microtome saw and polished for consecutive in situ chemical analysis. Initial analyses have been carried out using EMP collecting elemental concentration maps of Ca, Mg, Sr, S, and K. LA-MC-ICP-MS has been applied to simultaneously obtain 2D images of the distribution of boron isotopes ^10^B and ^11^B and carbon isotope ^12^C. Both EMP and LA-MC-ICP-MS have been applied in a multistep approach with respect to spatial resolution: (i) low-resolution overview scan of the entire sample section and (ii) high-resolution scan of an area selected from the overview scan for its high degree of spatial complexity. A detailed description of the analytical procedures is provided in the Supplementary Materials (S1: sample preparation; S2: EMP analyses; and S3: LA-MC-ICP-MS).

## Supplementary Materials

### This PDF file includes:

Supplementary Materials Tables S1 to S3 Figs. S1 to S7 References

## Notes

View/request a protocol for this paper from *Bio-protocol*.
