# Supplementary material for: Distinct fine-scale variations in calcification control revealed by high-resolution 2D boron laser images in the cold-water coral Lophelia pertusa
Source: Sci Adv. 2022 Mar 18;8(11):eabj4172. doi: 10.1126/sciadv.abj4172 (PMC8932653; doi:10.1126/sciadv.abj4172)
Supplement: Supplementary file 1 — Supplementary Materials Tables S1 to S3 Figs. S1 to S7 References [file sciadv.abj4172_sm.pdf]

Science Advances
AAAS

Supplementary Materials for

Distinct fine-scale variations in calcification control revealed by high-resolution 2D boron laser images in the cold-water coral *Lophelia pertusa*

Jan Fietzke* and Marlene Wall

*Corresponding author. Email: jfietzke@geomar.de

Published 18 March 2022, *Sci. Adv.* **8**, eabj4172 (2022)
DOI: 10.1126/sciadv.abj4172

This PDF file includes:

Supplementary Materials
Tables S1 to S3
Figs. S1 to S7
References

Supplementary Materials

S1 Sample preparation

A branch of the *Lophelia pertusa* sample had been embedded in epoxy resin (Araldite 2020). A ~1mm section was cut from the embedded sample using a microtome saw. One side of the sectioned sample was polished using Bühler "Micropolish" 6µm, 1µm and 0.05µm. Prior to EMP analyses the sample was coated with carbon.

S2 Electron microprobe analyses (EMP)

Electron microprobe analysis was carried out at GEOMAR Kiel, Germany, using a JEOL JXA 8200 "Superprobe". WDS (wavelength dispersive X-ray spectroscopy) elemental maps were acquired in two runs under the similar probe conditions (with the exception of the resolution; see Tab. S1):

1) Low resolution 2cm x 2cm overview map using 20µm probe diameter
2) High resolution 2mm x 2mm map of septum area using 5µm probe diameter, scanning the area marked by red rectangle in Figure 2a (main text).

Tab. S1: EMP measurement conditions

Acceleration Voltage	15kV
Beam current	100nA
Probe diameter (= pixel size)	20µm (low resolution run)<br>5µm (high resolution run)
Dwell time	50ms
Points	1000 x 1000 (low resolution run)<br>400 x 400 (high resolution run)
Accumulations	4
Elements (channel, crystal, spectral line)	1) Mg (TAPH, Kα1)<br>2) Ca (PETJ, Kα1)<br>3) Sr (TAP, Lα1)<br>4) S (PETH, Kα1)

Five standard materials (VG2, KAN1, celestine, calcite and dolomite) and spectral backgrounds (lower and upper) have been measured under identical probe conditions (10 x 10 pixels) yielding calibration parameters summarized in Tab. S2.

Tab.S2: EMP calibration (conc. [%] = ((cts/(I*t))-B)/A ; cts – counts, I – probe current, t – total dwell time per pixel, A – sensitivity parameter, B – background parameter

Element	A [cts/µA/ms/%]	B [cts/µA/ms]
Mg	19.741	1.900
Ca	9.296	0.734
Sr	6.328	1.355
S	15.221	0.502

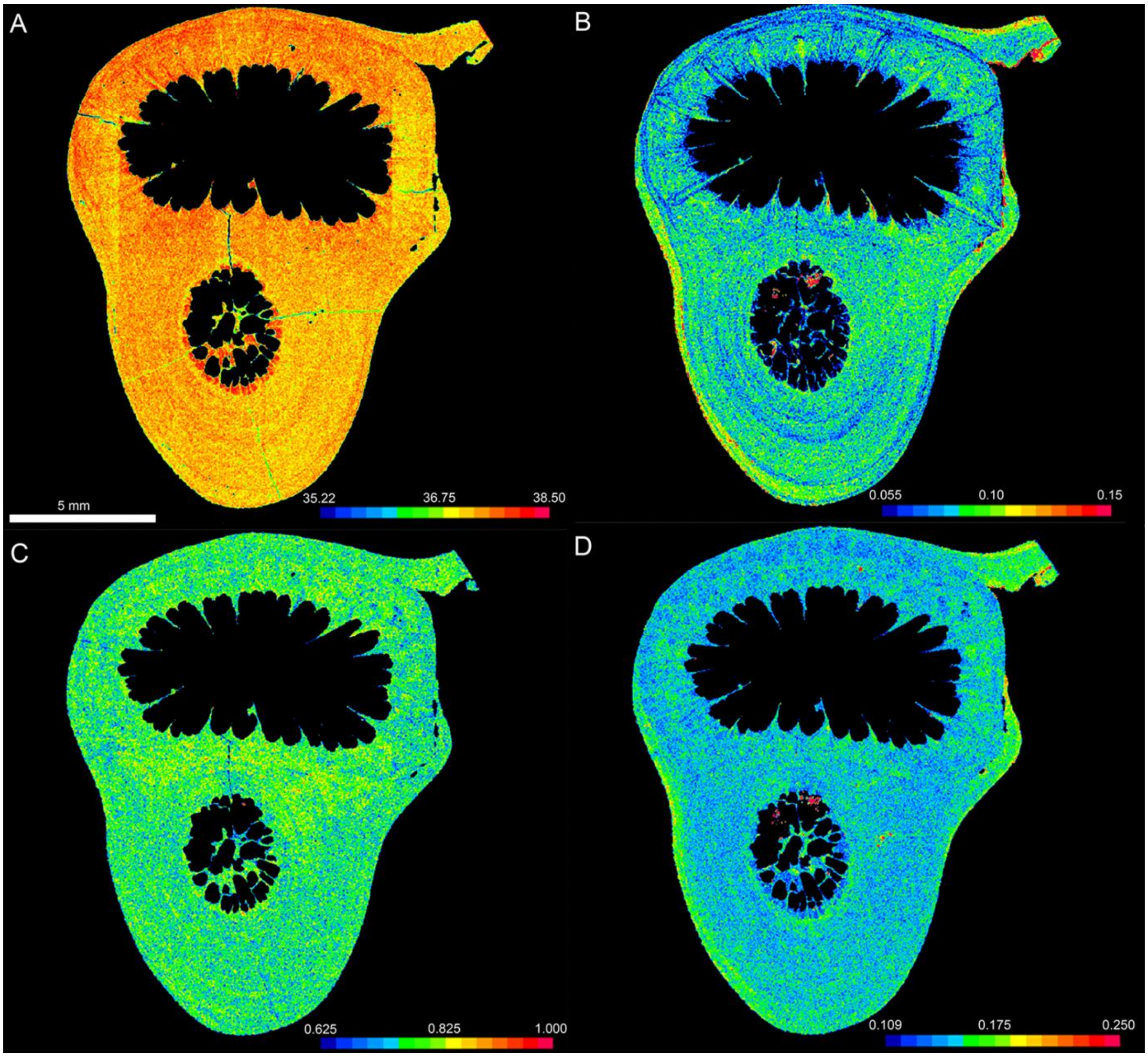

Fig. S1: Low resolution (20µm) EMP overview map of A) calcium, B) magnesium, C) strontium and D) sulphur. Maps depict element concentrations in weight%.

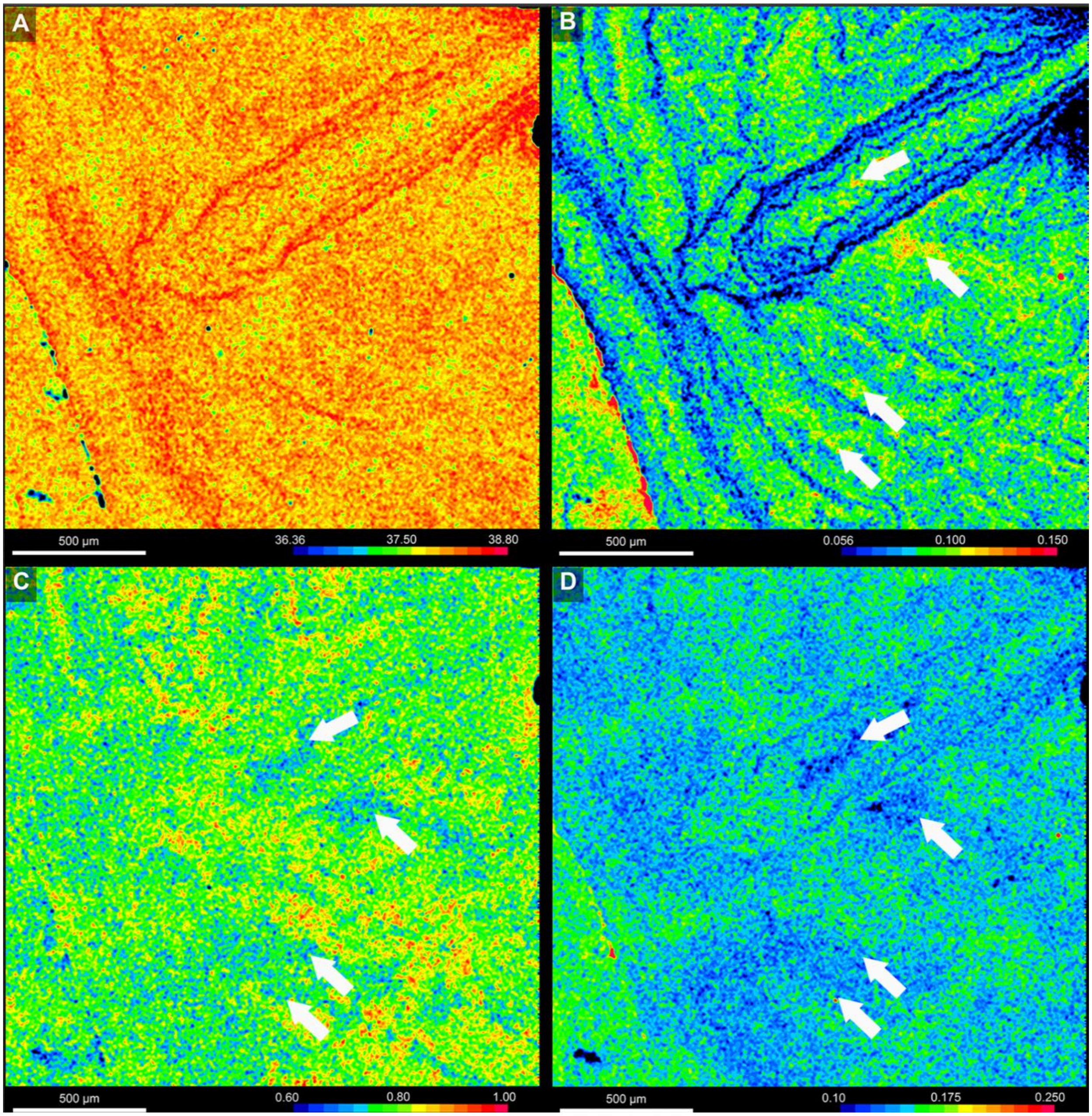

Fig. S2: High resolution (5µm) EMP overview map of A) calcium (Ca), B) magnesium, (Mg) C) strontium (Sr) and D) sulphur (S). Arrows indicate skeletal regions of distinct Mg composition and how they correlate with Sr and S composition. Maps depict element concentrations in weight%.

S3 Laser Ablation-MC-ICP-MS

After the sample had been analysed using electron microprobe it had been transferred to the LA-MC-ICP-MS for combined 2D boron and carbon isotope mapping. Again, a multi-step approach has been applied using 2 different settings of spatial resolution as well as two different ICP-plasma settings (cool and hot plasma) in the following order:

1) Two distinct measurements have been carried out using high sensitivity (cool plasma) conditions at a normalized Ar index NAI of 0.3 (*77*) and generated semi-quantitative images:

1.1) A low resolution overview map (using 100µm spot size and 50x80µm step size) has been collected to evaluate the overall distribution of B concentrations in the sample and select the area of interest for the high-resolution image approach.

1.2) A high resolution measurement (20µm spot size and 10x15µm step size), to create δ^{11}B and B concentration images of the area of interest which was accompanied by measurements of standard NIST-SRM610 for drift control and normalization.

2) To ensure accuracy of LA-MC-ICP-MS boron isotope results and provide fully-quantitative data the area used for high-resolution imaging had been re-run in low resolution under hot plasma conditions (NAI 14), together with five standards: NIST-SRM610 & 611, NBS951, Jcp-1 and Jct-1. The results have been used to normalize the high-resolution B image (see below).

Tab. S3: Operating conditions for LA-MC-ICP-MS (A: high sensitivity runs; B: high accuracy runs)

ICP rf power [W]	1100 (A); 1200 (B)
Gas flows [l/min] cool gas<br>auxiliary gas<br>sample gas<br>cell gas	1.7 (A & B)<br>1.1 (A); 1.2 (B)<br>0.8 (A); 0.4 (B)<br>0.75 (A: He); 0.6 (B: Ar)
NAI-normalized Ar index (*77*)	0.3 (A); 14 (B)
MA-matrix ion/Ar ratio (*77*)	<0.005 (A); <0.002 (B)
Laser fluence [J/cm²]	3 (A); 3.6 (B)
Repetition rate [Hz]	3 (A: low res)<br>8 (A: high res)<br>5 (B)
Laser spot size [µm]; distance between lines [µm]	100; 80 (A: low res & B)<br>20; 15 (A: high res)
Line scan speed [µm/s]; line length [µm]	50; 17500 (A: low res)<br>10; 1700 (A: high res)<br>50; 2500 (B)
Number of neighbouring lines	190 (A: low res)<br>102 (A: high res)<br>30 (B)
Full map repeats ("runs")	2 (A: low res)<br>10 (A: high res)<br>9 (B)
Integration time per data point [s]	1
Gas blank data collection period [s]	50
Typical background (gas blank) intensities [cps]	B10: <500 (A); <40 (B)<br>B11: <2000 (A); <200 (B)<br>C12: <2e8 (A); <1e7 (B)

LA-MC-ICP-MS analyses have been carried out using an "AXIOM" MC-ICP-MS (Thermo Fisher Sci., originally designed by VG) coupled to an ESI NWR UP193fx excimer laser ablation unit equipped with a large format cell (LFC). The analytical procedures closely

followed the previously published approach (*55*, *78–80*). Boron isotope (B10 and B11) signal intensities were measured using multi-ion-counting via 2 of the 3 AXIOM's channeltron ion counters. Simultaneously, C12 intensities have been recorded on a Faraday cup. The typical operation conditions are provided in Tab. S3.

C12 background intensities from the gas blank have been used to optimize the ion optics of the MC-ICP-MS. C12 intensities provide a good monitor for stability and sensitivity of the instrumentation. Tuning of the instrumentation focused on long-term stability.

Semi-quantitative low-resolution B concentration images (cool plasma)

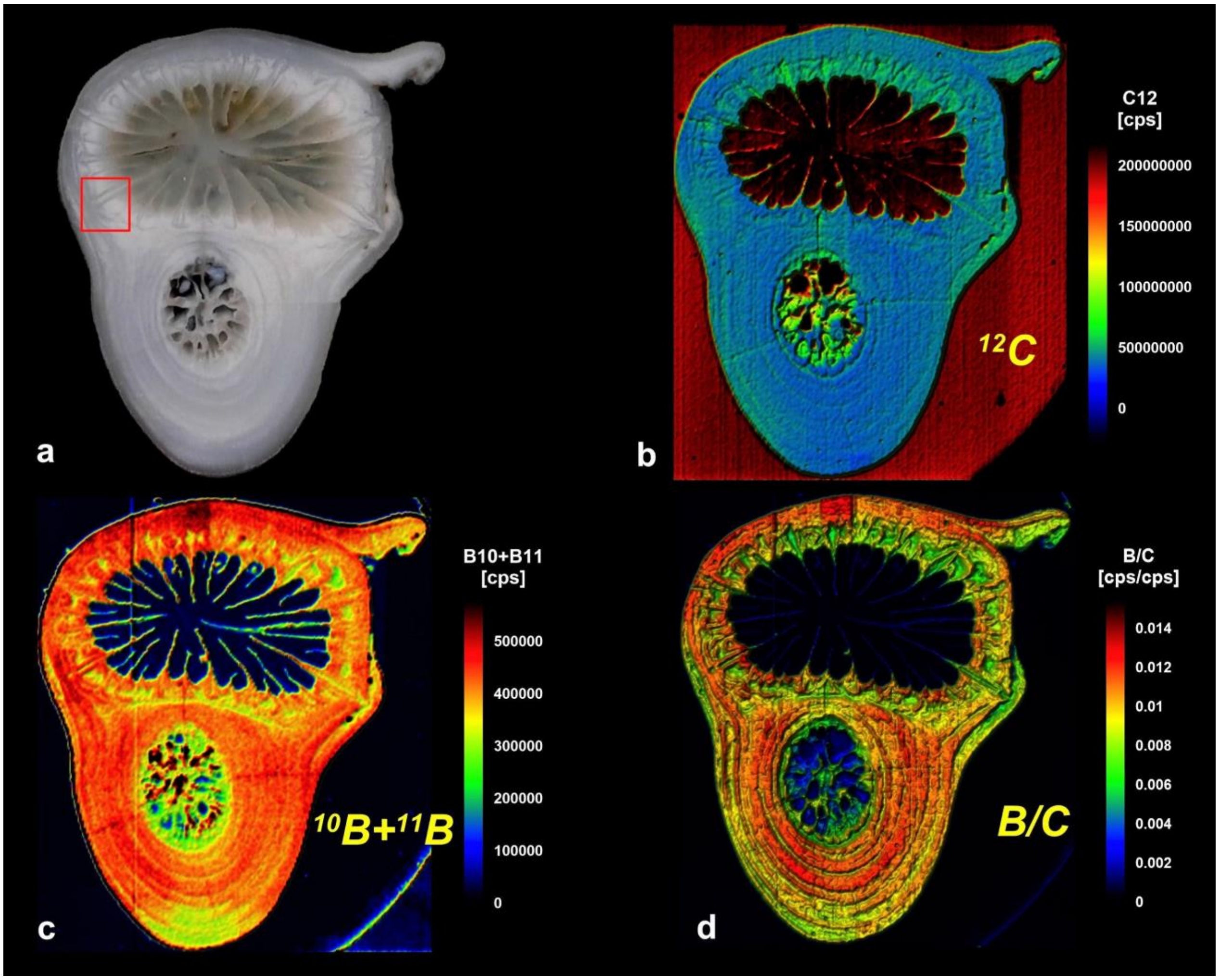

Fig. S3: *Lophelia pertusa* sample section (low resolution: laser spot diameter 100µm, step sizes 50µm vertical and 80µm horizontal). a) Optical image; red rectangle indicating the area covered by high resolution LA-MC-ICP-MS (Fig. 2c & d). b) Carbon (^{12}C) ion intensity image obtained by LA-MC-ICP-MS. High ^{12}C intensities (red) surrounding the sample belong to easily ablated epoxy resin used for embedding the sample. c) Boron (^{10}B+^{11}B) ion intensities obtained by LA-MC-ICP-MS. d) LA-MC-ICP-MS image of boron (^{10}B+^{11}B) normalized to carbon (^{12}C) ion intensity as a semi-quantitative measure of boron concentration distribution. Size of sampled area 17x15 mm^2.

The overview image analyses run at low resolution (100µm spot diameter, line scans, line spacing 80µm) did not include measurements of external standards for calibration. The only

purpose of this overview scans, besides pre-ablating the sample surface, had been to evaluate the internal variability and systematic distribution of boron within the section of the sample.

After background correction (based on the gas blank data collected before and after each line ablation) the total boron ion intensity (B10+B11) has been normalized to the C12 intensity. To improve the image quality the corresponding pixel data from both runs have been integrated for each isotope. Additionally, data smoothing has been applied, averaging two consecutive data points (two-point-running-mean, 50µm apart) within each ablation line data set. No smoothing of pixel data points between individual lines has been applied. The final images (produced using the software Golden Software "Surfer") are shown in Fig. S3 (b) C intensities and c) B10+B11 intensities and d) B/C intensity ratios).

High-resolution images (cool plasma)

The high-resolution image data have been run in a similar way but included 2 line-measurements of NIST-SRM610 glass standard at the beginning and after every set of 34 sample lines for drift control/correction. The whole area used for the high-resolution boron image consisted of 102 lines per single run. The image runs have been repeated 10 times.

Following the data acquisition, the raw intensities of the 10 runs have been integrated for each image pixel and each isotope and background corrected. For the ***B concentration maps*** the intensity ratios of (B10+B11)/C12 have been calculated and converted into B concentrations in µmol/mol following the same procedure as described in other studies (*50*, *80*). The high-resolution image (Fig. 3c main text) has been prepared using Golden Software "Surfer". No smoothing of data points has been applied for this image.

For the ***boron isotope high-resolution image*** (Fig. 3d main text) the background-corrected B10 and B11 intensities (integrated from all 10 runs) first had been smoothed (mean of 3x3 pixels, integrating an area of 45x30µm) that resulted in a reduction of image noise at acceptable cost of image resolution. The precision for each δ^{11}B image data point is mostly limited (as shown in previous papers e.g. Mayk et al. 2020 (*80*)) by counting statistics. Our typical B10 intensity was ~20.000-25.000 cps to limit the typical B11 intensity to ~100.000cps. The integration of 10 repeated runs reduces that noise level to ~2permil per pixel. The consecutive 3x3 pixel smoothing for the final δ^{11}B data ultimately reduces the noise level to ~0.7permil. For high B concentration areas our uncertainty for each image pixel in Figure 2d is ~1.5permil (2sd) and for low B concentration areas ~2permil (2sd).

Additionally, the image data have been recalibrated as outlined in the following section.

Calibration run of the high-resolution image (hot plasma)

Several recent studies reported issues with boron isotope data accuracy using LA-MC-ICP-MS (*81–83*). All three of those studies used a Thermo Neptune MC-ICP-MS and reported offsets in measured boron isotopes, which varied systematically with the sample's B/Ca ratio. Changing backgrounds from scattered ions (predominantly impacting ^{10}B) have been identified to be responsible for the observed systematic shifts towards apparently lighter

$\delta^{11}B$ values with lower B/Ca sample ratios. These observations resulted in the necessity to establish calibration curves for correcting for the $\delta^{11}B$ vs. (B/Ca) effect using a set of carbonate standards of known $\delta^{11}B$ and B/Ca, which allow accurate and precise data to be measured for unknown carbonate samples using the correction.

As shown in the study of Mayk et al. (*80*) when using the AXIOM MC-ICP-MS laser ablation B isotope data accuracy is primarily impacted by the plasma conditions/temperature, which can be determined by the normalized Ar index (NAI) as introduced by Fietzke & Frische (*77*). No comparable scattered ions effect is observed on the AXIOM MC-ICP-MS as evidenced by the off-peak baselines to ~1 cps for B10 and ~10 cps for B11. Nevertheless, the choice of plasma conditions significantly influences the matrix-tolerance. Matrix-specific-offsets occur between e.g. silicate (used as standards) and carbonate samples (and standards) resulting in a distortion of the reference frame, i.e. an offset in the normalization between carbonates and silicates. A comprehensive discussion of the nature of analytical offsets in B isotope LA-MC-ICP-MS is beyond the scope of this study. It deserves an entire separate analytical paper, which is being prepared.

Thus, to ensure B image data to be accurate the image area has been resampled in an additional ***hot plasma session*** (ICP under NAI=14) using the following 5 standards for calibration and quality control: NIST-SRM610 (silicate, glass), NIST-SRM611 (silicate, glass), NBS951 (boric acid, pellet), JCp-1 (carbonate, pellet) and JCt-1 (carbonate, pellet). The results of all standards agree with their accepted values (see Fig. S4).

The mean B isotope composition (integrated over the total image area) measured under hot plasma conditions displays an approx. +1.5 ‰ fractionation offset compared to the respective area mean measured in the high-resolution image cold plasma data acquisition (measured only relative to NIST-SRM610). The results of both sessions, i.e. high-resolution image data acquisition under low NAI and calibration under high NAI are displayed in Fig. S4. This fractionation offset has been accounted for by multiplying the individual pixel B11/B10 data of the high-resolution image by 1.00153, resulting in a mean B isotope composition of the image area in accord with the mean result of the calibration measurements. All boron isotope data are expressed in the typical delta notation ($\delta^{11}B$), as per mill difference of the [B11/B10] isotope abundances ratio of the sample relative to the [B11/B10] of the commonly used boric acid standard NBS951:

$$\delta^{11}B\ [‰] = (^{11}B/^{10}B)_{sample}/(^{11}B/^{10}B)_{NBS951}*1000-1000.$$

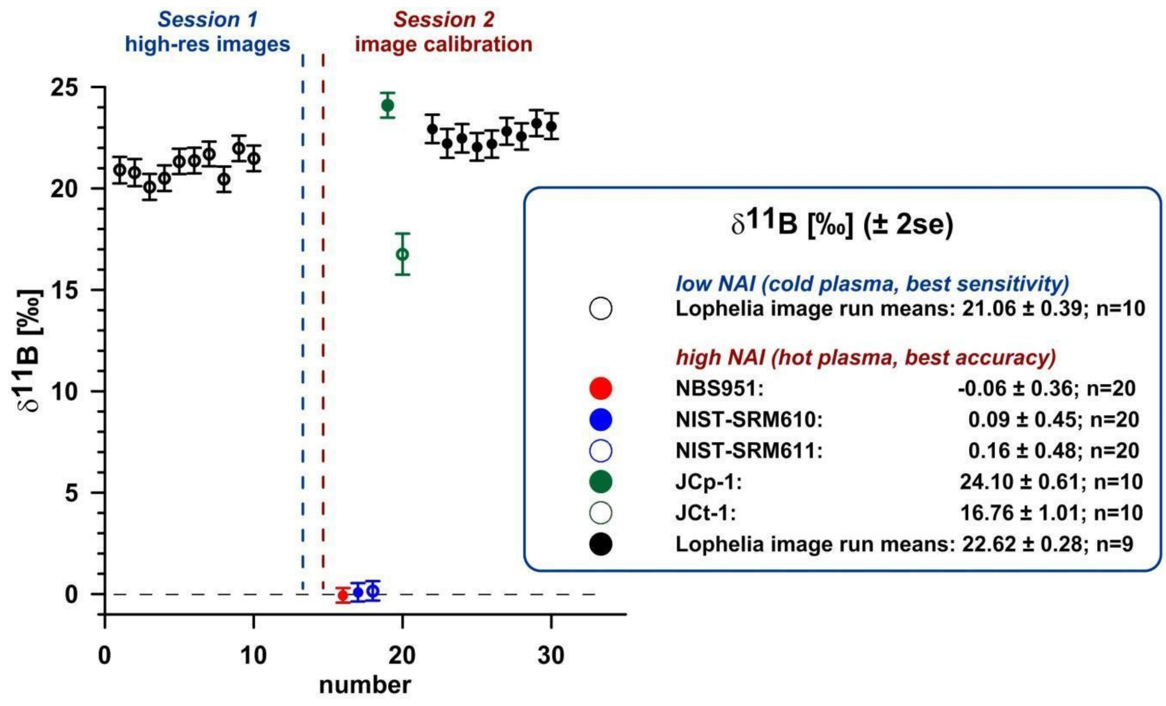

Fig. S4: Mean boron isotope composition of the area sampled for the high-resolution laser images. *Session1:* low NAI (cold plasma) for best sensitivity i.e. highest spatial resolution. 10 repeated full image runs, bracketed by NIST-SRM610 for drift control, δ-values calculated relative to NIST-SRM610. *Session2:* high NAI (hot plasma) for best accuracy. 9 repeated full image runs bracketed by 5 standard materials for accurate determination of the accurate and precise mean B isotope composition of the image area. Mean result used for re-calibrating the image data from high-sensitivity session.

Finally, we've evaluated if using low-NAI (maximum sensitivity) operational conditions result in systematic offsets in $\delta^{11}B$ depending on the B/Ca ratios of the respective carbonate as shown to be the case for Neptune MC-ICP-MS (*81-83*). Tuning the AXIOM MC-ICP-MS again for maximum sensitivity (NAI=0.3) we've analyzed JCp-1 and JCt-1. This resulted in $\delta^{11}B$ of 24.17 +- 0.24 ‰ (2se, n=24) for JCp-1 and 16.69 +- 0.44 ‰ (2se, n=24) for JCt-1, respectively.

The logic behind our normalization procedure is as follows:

1) Low-NAI measurement conditions provide the best sensitivity, allowing for the highest resolution in the image. As shown by the consistency of JCp-1 and JCt-1 data, even under such measurement conditions we do not observe a systematic trend in $\delta^{11}B$ for carbonates with differing B/Ca ratios in contrast to what has been reported in studies using Thermo Neptune MC-ICP-MS (*81-83*). This difference is most likely based on the significantly lower scattered ion background when using AXIOM MC-ICP-MS (off-peak baselines ~1cps (B10) and ~10cps (B11)).

2) Since no $\delta^{11}B$~(B/Ca)-trend could be detected, we conclude that the low-NAI $\delta^{11}B$ image data are not biased by the respective local differences in B/Ca. The data are internally consistent and the relative variations in $\delta^{11}B$ are accurately determined. What is missing at this point is the information on the accurate mean $\delta^{11}B$ value of the image.

3) Low-NAI conditions can be prone to matrix-specific offsets, making NIST-SRM610 as a silicate a biased point of reference for carbonates. However, the offset between carbonate and NIST-SRM610 is robust and stable (see Fig. S4, left panel) over the course of the 2-day session of initial image acquisition.

4) Using high-NAI conditions while being less sensitive, provides for accurate, matrix-independent determinations of $\delta^{11}B$ as shown by the consistency of all standards (silicates and carbonates alike) in Fig. S4 (right panel). Under these conditions, the mean $\delta^{11}B$ of the image area is accurately determined.

5) The accurate mean $\delta^{11}B$ is used to re-normalize the internally consistent initial $\delta^{11}B$ image.

Re-normalizing an internally consistent image to the accurate mean in our opinion does the job of establishing a fully accurate image. It preserves the internal variations and the accurate mean is achieved.

S4 Boron concentration distribution in theca thickening deposition growth layers

To test the potential for future B laser work in *L. pertusa* samples we have performed preliminary B/C LA-MC-ICP-MS maps in the thickening deposition growth layers forming the theca stem (see Fig. S5). Such a sample area would be of interest in the potential future use as an archive of sample growth over periods of years.

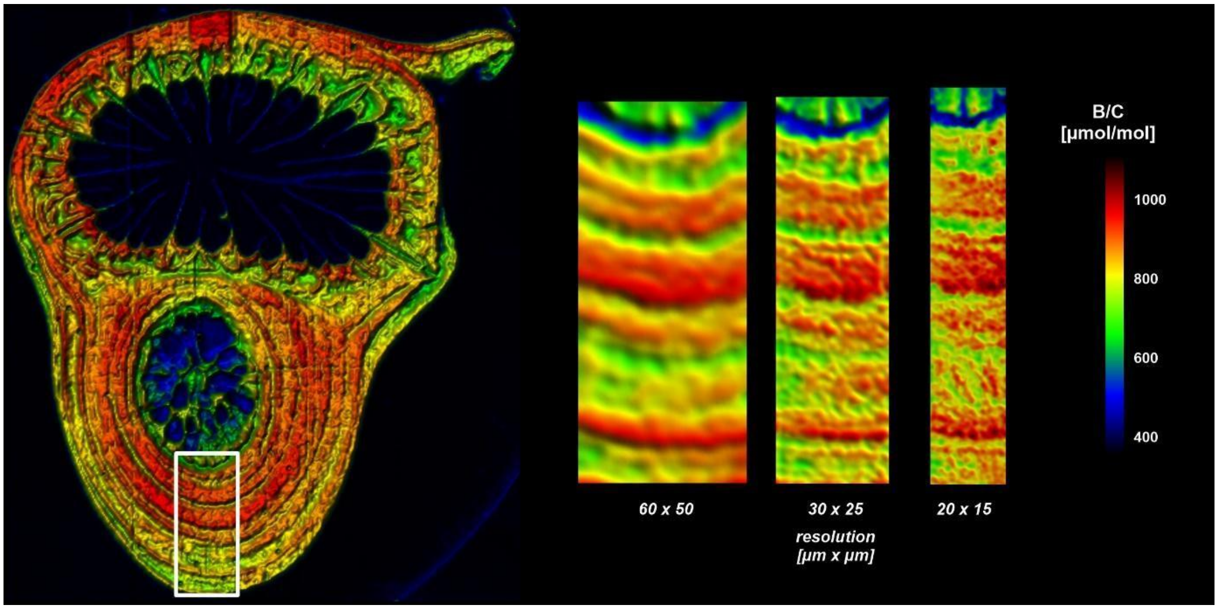

Fig. S5: B/C LA-MC-ICP-MS maps for the evaluation of the balance of analytical resolution and internal variability within the theca thickening deposition area. Left panel: B/C overview image from Fig. 2 (main text); resolution 80x50 µm. Right panel: 3 B/C maps from within the area marked by the rectangle in the overview image, gradually increasing the spatial resolution to 20x15 µm.

As can be seen in the resolution comparison (Fig. S5 right panel) a resolution of 30x25 µm appears to be a good compromise to capture the main features of internal B/C variability. The lowest B concentrations (~500 µmol/mol) are only found in the rapid accretion deposition part close to the center cavity of the stem (top of the high-resolution images). The concentric growth layers display systematic variations in B/C ranging from approx. 700 to 1000 µmol/mol. A more detailed investigation of the combined $\delta^{11}B$-B/C systematic in comparable skeletal parts from a variety of samples is planned for future studies.

S5 Calculation approach used for the ECM carbonate chemistry and related boron parameters in aragonite Fig. 3 (main text)

To evaluate the laser boron data (concentration and isotope composition) we test the concept of the calcifying fluid being derived from seawater and the organism exerting control only on the carbonate system parameters pH and DIC increasing saturation state (Ω) to precipitate new skeletal aragonite. The grid in Fig. 3 (main text) represents the composition of aragonite that would form under different sets of pH and DIC, carrying the boron isotope composition of the borate ion (pH-dependent) and a B/C ratio based on the K_D (pH- and DIC dependent) of borate/carbonate ion in aragonite vs. solution $(B/C)_{aragonite}=K_D*(B(OH)^{4-}/CO_3^{2-})_{solution}$.

As input parameters we use the condition of the local seawater from Büscher et al. (2017) (*76*):

- pH: 8.0 (total scale)
- DIC: 2150 µmol/kg
- temperature: 7.6-7.9 °C
- salinity: 35

These parameters result in a boric acid equilibrium constant pK_B of 8.82 (after (*30*)). The carbonic acid equilibrium constants are calculated (using the mean temperature and salinity) as pK_{S1}=6.023 and pK_{S2}=9.246 (*84*). For bulk seawater a boron isotope composition of 39.61 ‰ (*34*) and a boron isotope fractionation factor of 27.2 ‰ (*32*) are applied.

The concentrations of calcium and boron are set at 10.4 mMol/l and 0.436 mMol/l, respectively (*85*).

K_D is calculated adapting the equation provided in McCulloch et al. (*3*) (based on the data of Holcomb et al. (*35*)): $K_D=2.97e-3*\exp(-0.0202*[H^+])$.

S6 *Lophelia pertusa* skeletal structure and zonation

To further illustrate the skeletal heterogeneity and distribution of different skeletal zones additional transverse sections were taken in the same *Lophelia* specimen (see Fig. S6). The zonation follows the characterization in Stolarski (*64*) for *Desmophyllum dianthus* and the differentiation in three zones in terms of light-transparency properties: (1) rapid accretion deposits (RAD), a zone of approx. 15-20 µm width and of dark brown coloration, (2) zone of thickening deposits with brownish zonal coloration – lighter than RAD and (3) zone of transparent, nearly colorless thickening deposits.

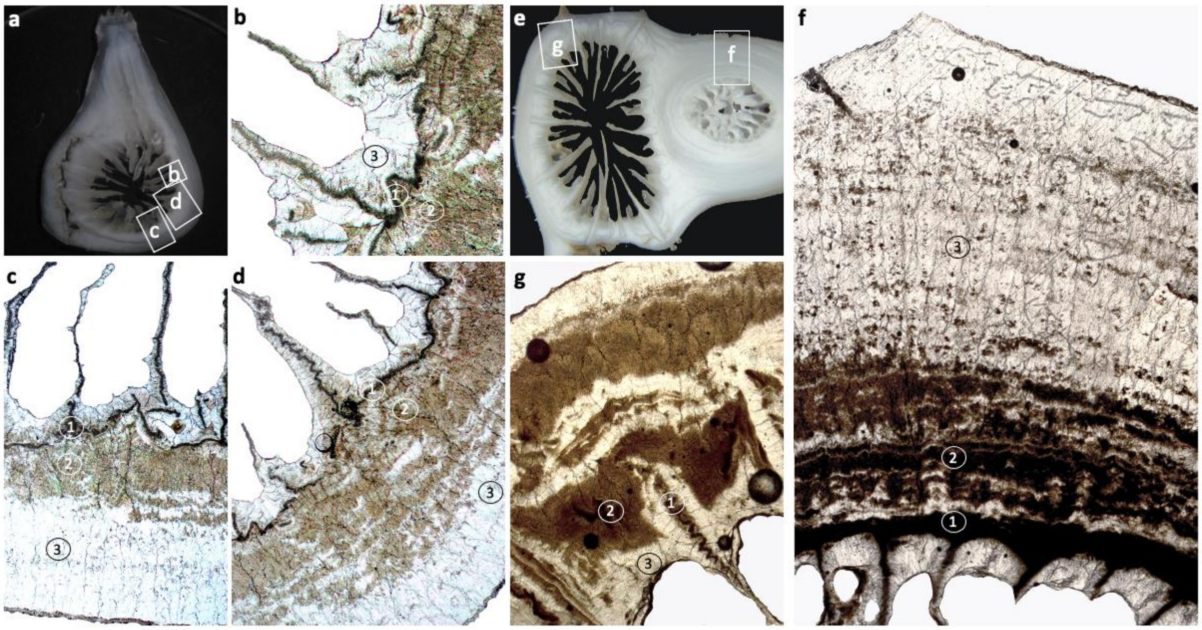

Fig. S6: Additional light and transmission light microscopy images of polished transversal section from the same *Lophelia* specimen. (a,e) Light microscopy image overview of different sections cutting different polyps of the same colony. b-d & f-g) Transverse polished section of septa and theca in transmission light microscopy showing dark-brown zones of septal as well as septo-thecal rapid accretion fronts (1) and theca thickening deposits (2) that clearly separates from bright areas of thickening deposits (3).

S7 Organics' contribution to skeletal boron

Organically bound B cannot be the main cause for the observed δ^{11}B range. Such a scenario would require large variations in organic content of the skeleton and an extremely light boron isotope organics endmember. We do not observe large variations in Ca and S (Ca conc.: 38.0 ± 0.3 wt%, S conc. 0.15 ± 0.02 wt%; see suppl. material S2 and Fig. S1 and S2) indicating the overwhelming dominance of carbonate. Any binary mixing scenario (carbonate vs. organics) would require the organics endmember to be extremely light in B isotopes and unrealistically contribute a negative amount of B to "dilute" the B concentration dominated by the carbonate endmember. Additionally, the agreement of our HH-B cluster data with published *L. pertusa* theca wall boron data (measured conventionally after removal of organic material) rules out major impacts by organics. Significant boron contributions from sources other than seawater e.g. nutrition can also be excluded since the variability observed in our sample is clearly linked to structural pattern of skeletal growth, whereas a strict dependency of the coral's structural development on a particular diet appears not reasonable.

S8 Potential temporal transition between growth modes

Within the results we mention a transition between the two endmembers of B composition (LL-B and HH-B) that align with areas marked in Figure 1 with (2) (see Fig. S7a). With our imaging approach we can with confidence link our observation spatially but less temporally since we do not know the rates of formation of the different skeletal parts. However, spatial data also have a temporal context and temporal information is reflected in these images. We are going to use this transition area to put our model (Fig. 4) in a more temporal context. It is certainly possible that the EMZ is initiated by a pulse of the LL-B calcification mode that overwhelms the other pathways present during calcification and later the other pathways take over by gradually shifting towards the HH-B mode of calcification (Fig. S7b). We outlined that the skeletal signature is a matter of I and O of individual transport processes and the dominating transport defines the signature. This already implies evolution of processes and the ability to gradual shifts. For instance, during LL-B formation PMCA may not be inactive, but not dominating (Fig. S7b). We also suggest that LL-B and HH-B mainly differ in BAT activity but not PMCA. Which also provides some temporal aspects like PMCA that may be constantly active while BAT shows clear differences in activity (which we also discuss based on gene expression studies in tropical corals). Within the transition area the measured B systematics suggest that pathways controlling B isotopic conditions persist longer compared to pathways controlling B concentration. Thus, restricted seawater leakage and BAT activity still need to be in play. The generation of the B concentration is much more dependent on DIC dynamics. These interdependencies restrict our ability to pinpoint the underlying key pathways and need further investigations outlined below in the section on C and O isotope signals. Overall, we did not want to overemphasize the temporal aspects since information on rates as well as on activity of certain transporters are currently missing. As suggested above it is possible that calcification is initiated by a strong pulse of LL-B or BAT activity but this may require a stronger pulse compared to a more prolonged activity at a lower rate for the time frame required to build EMZ. Similarly, temporal differences in calcification mode will also affect other transport pathways e.g. CO_2 diffusion and thus, other element or isotope signatures that need to be considered in the future.

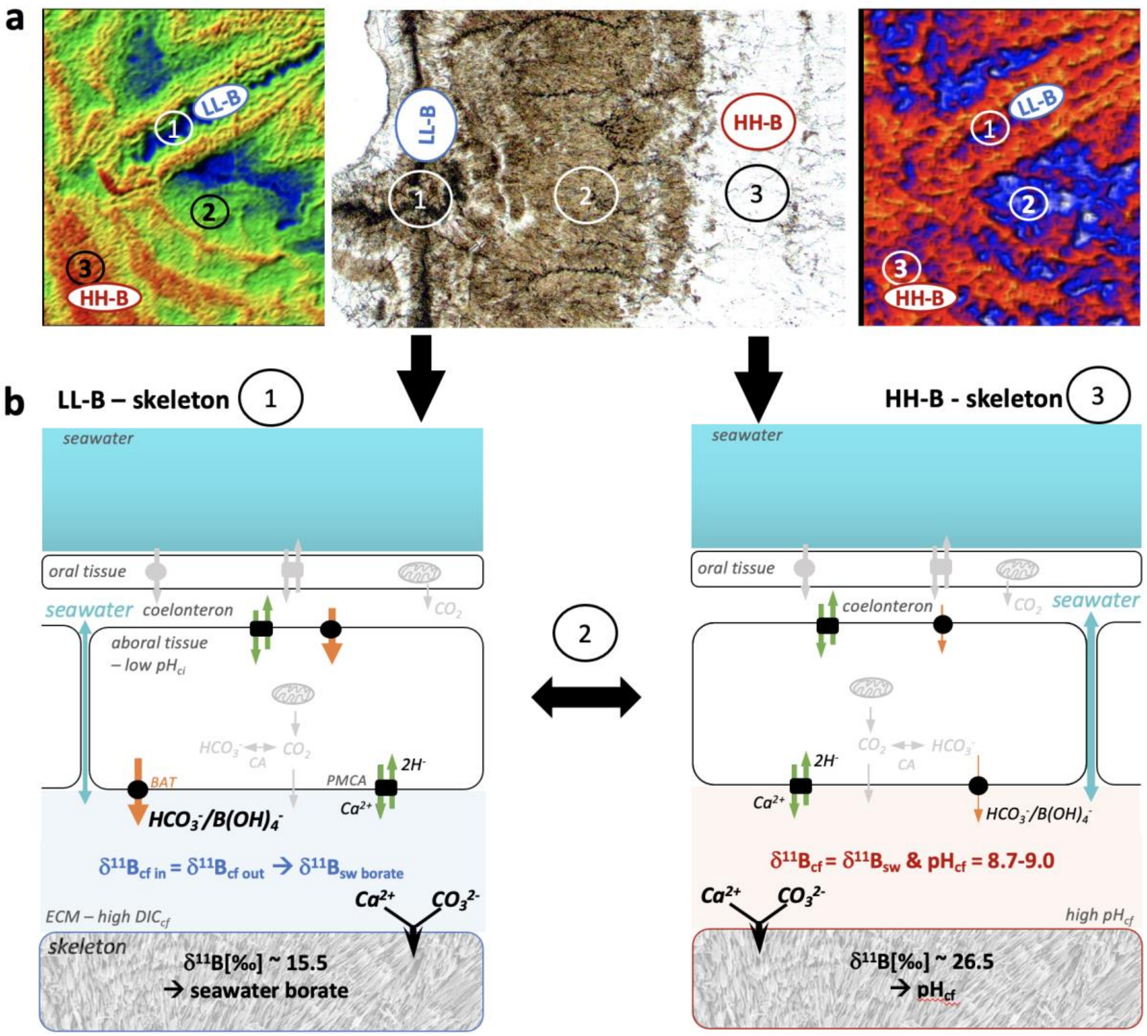

Fig S7: Proposed mode of calcification in a temporal context and focus on the transition area (2) from LL-B (1) to HH-B (3). a) Spatial distribution of boron concentration and δ^{11}B and how it links to the skeletal structure. b) Temporal differences in calcification mode with the two extreme end-members and potential processes during transition. For the transition area (2) low d11B values (comparable to LL-B) indicate the still dominating role of BAT as the transport pathway of isotopically light seawater borate ions into the ECM. That said, the B concentrations gradually increase from the low (LL-B) towards the lower end of the high (HH-B) composition. We take this observation as indication of a small imbalance in the DIC I and O fluxes, resulting in a gradual ingrowth of B in the ECM. We cannot quantify this, since the precise knowledge of all the relevant transport rates (incl. their temporal variability) is not yet existing.

S9 Relevance and consequences for skeletal C and O isotopic signature

Our focus in this study was on high resolution B systematics in corals and which modifications of the to date proposed calcification models are required to generate such signal. We focused on – in our perspective - key pathways that mainly affect B systematics namely PMCA, BAT and seawater leakage. Yet they are not independent of other relevant processes and modification of these pathways will have consequences for other transport processes and thus, affect other element and isotope ratios of the skeleton. Two critical isotope systems oxygen and carbon on the one hand provide additional support but also indicate inconsistencies – in particular for δ^{11}B and δ^{13}C studies no unifying mechanism exists to date (*72*). While we propose that borate co-transport via BAT distorts the signal

within the EMZ and is a main driver, this can affect the skeletal $\delta^{13}C$ signal. However, this does not mean that the BAT rates are necessarily very high. More important is a restricted seawater transport and thus, heavier $\delta^{11}B$ from seawater cannot reach the site of calcification. At the extreme end - a full shut down of the paracellular pathway - the bicarbonate transport and hence, borate co-transport, can be very low and bicarbonate transport is not the main source for DIC and thus may have restricted effect on $\delta^{13}C$. For the latter, the main DIC source can be from CO2 diffusion that supports a depleted $\delta^{13}C$ as observed for EMZ (*73*). Thus, from a $\delta^{13}C$ perspective BAT may not be the predominant process. To address this issue further studies on DIC transport in corals are desirable as well as $\delta^{13}C$ and $\delta^{18}O$ images with the same spatial resolution as the $\delta^{11}B$ and B/C images. We suggest some possibilities to overcome existing inconsistencies between isotopic systematics but they are far from being complete and a full model cannot be derived in this paper. We are certain that our proposed pathways will provide novel aspects that can help to improve existing models. Future research needs to address the missing pieces and provide better constraints for required rate estimates before we will be in the position to provide a fully-quantitative model.

REFERENCES AND NOTES

1. D. Allemand, É. Tambutté, D. Zoccola, S. Tambutté, Coral Calcification, Cells to Reefs, in *Coral Reefs: An Ecosystem in Transition*, Z. Dubinsky, N. Stambler, Eds. (Springer, Dordrecht, 2011), pp. 119–150.

2. D. Bhattacharya, S. Agrawal, M. Aranda, S. Baumgarten, M. Belcaid, J. L. Drake, D. Erwin, S. Foret, R. D. Gates, D. F. Gruber, B. Kamel, M. P. Lesser, O. Levy, Y. J. Liew, M. MacManes, T. Mass, M. Medina, S. Mehr, E. Meyer, D. C. Price, H. M. Putnam, H. Qiu, C. Shinzato, E. Shoguchi, A. J. Stokes, S. Tambutté, D. Tchernov, C. R. Voolstra, N. Wagner, C. W. Walker, A. P. M. Weber, V. Weis, E. Zelzion, D. Zoccola, P. G. Falkowski, Comparative genomics explains the evolutionary success of reef-forming corals. *eLife* **5**, e13288 (2016).

3. M. T. McCulloch, J. P. D'Olivo, J. Falter, M. Holcomb, J. A. Trotter, Coral calcification in a changing world and the interactive dynamics of pH and DIC upregulation. *Nat. Commun.* **8**, 15686 (2017).

4. A. A. Venn, E. Tambutte, M. Holcomb, D. Allemand, S. Tambutte, Live tissue imaging shows reef corals elevate pH under their calcifying tissue relative to seawater. *PLOS ONE* **6**, e20013 (2011).

5. A. A. Venn, E. Tambutté, M. Holcomb, J. Laurent, D. Allemand, S. Tambutté, Impact of seawater acidification on pH at the tissue – skeleton interface and calcification in reef corals. *Proc. Natl. Acad. Sci. U.S.A.* **110**, 1634–1639 (2013).

6. D. S. Sevilgen, A. A. Venn, M. Y. Hu, E. Tambutté, D. De Beer, V. Planas-Bielsa, S. Tambutté, Full in vivo characterization of carbonate chemistry at the site of calcification in corals. *Sci. Adv.* **5**, eaau7447 (2019).

7. A. T. Marshall, P. L. Clode, R. Russell, K. Prince, R. Stern, Electron and ion microprobe analysis of calcium distribution and transport in coral tissues. *J. Exp. Biol.* **210**, 2453–63 (2007).

8. A. A. Venn, E. Tambutté, S. Lotto, D. Zoccola, D. Allemand, S. Tambutté, Imaging intracellular pH in a reef coral and symbiotic anemone. *Proc. Natl. Acad. Sci. U.S.A.* **106**, 16574–16579 (2009).

9. P. L. Clode, A. T. Marshall, Low temperature X-ray microanalysis of calcium in a scleractinian coral: Evidence of active transport mechanisms. *J. Exp. Biol.* **205**, 3543–3552 (2002).

10. S. Tambutté, E. Tambutté, D. Zoccola, N. Caminiti, S. Lotto, A. Moya, D. Allemand, J. Adkins, Characterization and role of carbonic anhydrase in the calcification process of the azooxanthellate coral *Tubastrea aurea*. *Mar. Biol.* **151**, 71–83 (2007).

11. A. A. Venn, C. Bernardet, A. Chabenat, E. Tambutté, S. Tambutté, Paracellular transport to the coral calcifying medium: Effects of environmental parameters. *J. Exp. Biol.* **223**, jeb227074 (2020).

12. E. Tambutte, S. Tambutte, N. Segonds, D. Zoccola, A. Venn, J. Erez, D. Allemand, Calcein labelling and electrophysiology: Insights on coral tissue permeability and calcification. *Proc. R. Soc. B Biol. Sci.* **279**, 19–27 (2012).

13. J. F. Adkins, E. A. Boyle, W. B. Curry, A. Lutringer, Stable isotopes in deep-sea corals and a new mechanism for "vital effects". *Geochem. Cosmochim. Acta* **67**, 1129–1143 (2003).

14. A. C. Gagnon, J. F. Adkins, J. Erez, Seawater transport during coral biomineralization. *Earth Planet. Sci. Lett.* **329-330**, 150–161 (2012).

15. T. A. McConnaughey, J. F. Whelan, Calcification generates protons for nutrient and bicarbonate uptake. *Earth Sci. Rev.* **42**, 95–117 (1997).

16. D. Zoccola, E. Tambutté, E. Kulhanek, S. Puverel, J.-C. Scimeca, D. Allemand, S. Tambutté, Molecular cloning and localization of a PMCA P-type calcium ATPase from the coral *Stylophora pistillata*. *Biochim. Biophys. Acta* **1663**, 117–126 (2004).

17. T. M. DeCarlo, S. Comeau, C. E. Cornwall, M. T. McCulloch, Coral resistance to ocean acidification linked to increased calcium at the site of calcification, *Proc. R. Soc. B Biol. Sci.* **285**, 20180564 (2018).

18. X. Wang, D. Zoccola, Y. J. Liew, E. Tambutte, G. Cui, D. Allemand, S. Tambutte, M. Aranda, The evolution of calcification in reef-building corals. *Mol. Biol. Evol.* **38**, 3543–3555 (2021).

19. P. Furla, I. Galgani, I. Durand, D. Allemand, Sources and mechanisms of inorganic carbon transport for coral calcification and photosynthesis. *J. Exp. Biol.* **203**, 3445–57 (2000).

20. S. Benazet-Tambutte, D. Allemand, J. Jaubert, Permeability of the oral epithelial layers in cnidarians. *Mar. Biol.* **126**, 43–53 (1996).

21. D. Zoccola, P. Ganot, A. Bertucci, N. Caminiti-Segonds, N. Techer, C. R. Voolstra, M. Aranda, E. Tambutté, D. Allemand, J. R. Casey, S. Tambutté, Bicarbonate transporters in corals point towards a key step in the evolution of cnidarian calcification. *Sci. Rep.* **5**, 9983 (2015).

22. L. Addadi, S. Raz, S. Weiner, Taking advantage of disorder: Amorphous calcium carbonate and its roles in biomineralization. *Adv. Mater.* **15**, 959–970 (2003).

23. Y. Politi, D. R. Batchelor, P. Zaslansky, B. F. Chmelka, J. C. Weaver, I. Sagi, S. Weiner, L. Addadi, Role of magnesium ion in the stabilization of biogenic amorphous calcium carbonate: A structure−function investigation. *Chem. Mater.* **22**, 161–166 (2010).

24. J. L. Drake, T. Mass, J. Stolarski, S. Von Euw, B. van de Schootbrugge, P. G. Falkowski, How corals made rocks through the ages. *Glob. Chang. Biol.* **26**, 31–53 (2020).

25. C. Y. Sun, C. A. Stifler, R. V. Chopdekar, C. A. Schmidt, G. Parida, V. Schoeppler, B. I. Fordyce, J. H. Brau, T. Mass, S. Tambutté, P. U. P. A. Gilbert, From particle attachment to space-filling coral skeletons. *Proc. Natl. Acad. Sci. U.S.A.* **117**, 30159–30170 (2020).

26. N. G. Hemming, G. N. Hanson, Boron isotopic composition and concentration in modern marine carbonates. *Geochim. Cosmochim. Acta* **56**, 537–543 (1992).

27. M. Pagani, D. Lemarchand, A. Spivack, J. Gaillardet, A critical evaluation of the boron isotope-pH proxy: The accuracy of ancient ocean pH estimates. *Geochim. Cosmochim. Acta* **69**, 953–961 (2005).

28. B. Hönisch, N. G. Hemming, Surface ocean pH response to variations in pCO_2 through two full glacial cycles. *Earth Planet. Sci. Lett.* **236**, 305–314 (2005).

29. N. Allison, I. Cohen, A. Finch, J. Erez, A. W. Tudhope, Corals concentrate dissolved inorganic carbon to facilitate calcification. *Nat. Commun.* **5**, 5741 (2014).

30. A. G. Dickson, Thermodynamics of the dissociation of boric acid in synthetic seawater from 273.15 to 318.15 K. *Deep Sea Res. A.* **37**, 755–766 (1990).

31. R. E. Zeebe, A. Sanyal, J. D. Ortiz, D. A. Wolf-Gladrow, A theoretical study of the kinetics of the boric acid–borate equilibrium in seawater. *Mar. Chem.* **73**, 113–124 (2001).

32. K. Klochko, A. J. Kaufman, W. Yao, R. H. Byrne, J. A. Tossell, Experimental measurement of boron isotope fractionation in seawater. *Earth Planet. Sci. Lett.* **248**, 276–285 (2006).

33. K. Klochko, G. D. Cody, J. A. Tossell, P. Dera, A. J. Kaufman, Re-evaluating boron speciation in biogenic calcite and aragonite using ^{11}B MAS NMR. *Geochim. Cosmochim. Acta* **73**, 1890–1900 (2009).

34. G. L. Foster, P. A. E. Pogge von Strandmann, J. W. B. Rae, Boron and magnesium isotopic composition of seawater. *Geochem. Geophys. Geosyst.* **11**, Q08015 (2010).

35. M. Holcomb, T. M. DeCarlo, G. A. Gaetani, M. McCulloch, Factors affecting B/Ca ratios in synthetic aragonite. *Chem. Geol.* **437**, 67–76 (2016).

36. T. M. Decarlo, M. Holcomb, M. T. McCulloch, Reviews and syntheses: Revisiting the boron systematics of aragonite and their application to coral calcification. *Biogeosciences* **15**, 2819–2834 (2018).

37. B. Hönisch, N. Hemming, A. Grottoli, A. Amat, G. Hanson, J. Bijma, Assessing scleractinian corals as recorders for paleo-pH: Empirical calibration and vital effects. *Geochim. Cosmochim. Acta* **68**, 3675–3685 (2004).

38. S. Krief, E. J. Hendy, M. Fine, R. Yam, A. Meibom, G. L. Foster, A. Shemesh, Physiological and isotopic responses of scleractinian corals to ocean acidification. *Geochim. Cosmochim. Acta* **74**, 4988–5001 (2010).

39. M. Holcomb, A. Venn, E. Tambutté, S. Tambutté, D. Allemand, J. Trotter, M. McCulloch, Coral calcifying fluid pH dictates response to ocean acidification. *Sci. Rep.* **4**, 5207 (2014).

40. J. Trotter, P. Montagna, M. McCulloch, S. Silenzi, S. Reynaud, G. Mortimer, S. Martin, C. Ferrier-Pagès, J.-P. Gattuso, R. Rodolfo-Metalpa, Quantifying the pH 'vital effect' in the temperate zooxanthellate coral *Cladocora caespitosa*: Validation of the boron seawater pH proxy. *Earth Planet. Sci. Lett.* **303**, 163–173 (2011).

41. M. McCulloch, J. Trotter, P. Montagna, J. Falter, R. Dunbar, A. Freiwald, G. Försterra, M. López Correa, C. Maier, A. Rüggeberg, M. Taviani, Resilience of cold-water scleractinian corals to ocean acidification: Boron isotopic systematics of pH and saturation state up-regulation. *Geochim. Cosmochim. Acta* **87**, 21–34 (2012).

42. E. Anagnostou, K. Huang, C. You, E. L. Sikes, R. M. Sherrell, Evaluation of boron isotope ratio as a pH proxy in the deep sea coral *Desmophyllum dianthus*: Evidence of physiological pH adjustment. *Earth Planet. Sci. Lett.* **349-350**, 251–260 (2012).

43. J. Raddatz, V. Liebetrau, J. Trotter, A. Rüggeberg, S. Flögel, W. C. Dullo, A. Eisenhauer, S. Voigt, M. McCulloch, Environmental constraints on Holocene cold-water coral reef growth off Norway: Insights from a multiproxy approach. *Paleoceanography* **31**, 1350–1367 (2016).

44. C. Rollion-Bard, M. Chaussidon, C. France-Lanord, pH control on oxygen isotopic composition of symbiotic corals. *Earth Planet. Sci. Lett.* **215**, 275–288 (2003).

45. D. Blamart, C. Rollion-Bard, A. Meibom, J.-P. Cuif, A. Juillet-Leclerc, Y. Dauphin, Correlation of boron isotopic composition with ultrastructure in the deep-sea coral *Lophelia pertusa*: Implications for biomineralization and paleo-pH. *Geochem. Geophys. Geosyst.* **8**, Q12001 (2007).

46. M. McCulloch, J. Falter, J. Trotter, P. Montagna, Coral resilience to ocean acidification and global warming through pH up-regulation. *Nat. Clim. Chang.* **2**, 623–627 (2012).

47. V. Schoepf, M. T. McCulloch, M. E. Warner, S. J. Levas, Y. Matsui, M. D. Aschaffenburg, A. G. Grottoli, Short-term coral bleaching is not recorded by skeletal boron isotopes. *PLOS ONE* **9**, e112011 (2014).

48. F. A. Al-Horani, S. M. Al-Moghrabi, D. de Beer, Microsensor study of photosynthesis and calcification in the scleractinian coral, *Galaxea fascicularis*: Active internal carbon cycle. *J. Exp. Mar. Bio. Ecol.* **288**, 1–15 (2003).

49. K. L. Barott, S. O. Perez, L. B. Linsmayer, M. Tresguerres, Differential localization of ion transporters suggests distinct cellular mechanisms for calcification and photosynthesis between two coral species. *Am. J. Physiol. Regul. Integr. Comp. Physiol.* **309**, R235–R246 (2015).

50. M. Wall, J. Fietzke, E. D. Crook, A. Paytan, Using B isotopes and B/Ca in corals from low saturation springs to constrain calcification mechanisms. *Nat. Commun.* **10**, 3580 (2019).

51. V. Schoepf, C. P. Jury, R. J. Toonen, M. T. McCulloch, Coral calcification mechanisms facilitate adaptive responses to ocean acidification. *Proc. Biol. Sci.* **284**, 20172117 (2017).

52. A. Meibom, Distribution of magnesium in coral skeleton. *Geophys. Res. Lett.* **31**, 10.1029/2004GL021313 (2004).

53. J.-P. Cuif, Y. Dauphin, J. Doucet, M. Salome, J. Susini, XANES mapping of organic sulfate in three scleractinian coral skeletons. *Geochim. CosmochIm. Acta* **67**, 75–83 (2003).

54. T. B. Chalk, C. D. Standish, C. D'Angelo, K. D. Castillo, J. A. Milton, G. L. Foster, Mapping coral calcification strategies from in situ boron isotope and trace element measurements of the tropical coral *Siderastrea siderea*. *Sci. Rep.* **11**, 472 (2021).

55. J. Fietzke, F. Ragazzola, J. Halfar, H. Dietze, L. C. Foster, T. H. Hansteen, A. Eisenhauer, R. S. Steneck, Century-scale trends and seasonality in pH and temperature for shallow zones of the Bering Sea. *Proc. Natl. Acad. Sci. U.S.A.* **112**, 2960–2965 (2015).

56. A. M. Addamo, A. Vertino, J. Stolarski, R. García-Jiménez, M. Taviani, A. Machordom, Merging scleractinian genera: The overwhelming genetic similarity between solitary *Desmophyllum* and colonial *Lophelia*. *BMC Evol. Biol.* **16**, 108 (2016).

57. M. Wall, F. Ragazzola, L. C. Foster, A. Form, D. N. Schmidt, pH up-regulation as a potential mechanism for the cold-water coral *Lophelia pertusa* to sustain growth in aragonite undersaturated conditions. *Biogeosciences* **12**, 6869–6880 (2015).

58. C. Rollion-Bard, D. Blamart, J.-P. Cuif, A. Juillet-Leclerc, Microanalysis of C and O isotopes of azooxanthellate and zooxanthellate corals by ion microprobe. *Coral Reefs* **22**, 405–415 (2003).

59. J. Raddatz, V. Liebetrau, A. Rüggeberg, E. Hathorne, A. Krabbenhöft, A. Eisenhauer, F. Böhm, H. Vollstaedt, J. Fietzke, M. López Correa, A. Freiwald, W. C. Dullo, Stable Sr-isotope, Sr/Ca, Mg/Ca, Li/Ca and Mg/Li ratios in the scleractinian cold-water coral *Lophelia pertusa*. *Chem. Geol.* **352**, 143–152 (2013).

60. H. Jurikova, V. Liebetrau, J. Raddatz, J. Fietzke, J. Trotter, A. Rocholl, S. Krause, M. McCulloch, A. Rüggeberg, A. Eisenhauer, Boron isotope composition of the cold-water coral *Lophelia pertusa* along the Norwegian margin: Zooming into a potential pH-proxy by combining bulk and high-resolution approaches. *Chem. Geol.* **513**, 143–152 (2019).

61. T. M. DeCarlo, H. Ren, G. Farfan, The origin and role of organic matrix in coral calcification: Insights from comparing coral skeleton and abiogenic aragonite. *Front. Mar. Sci.* **5**, 170 (2018).

62. M. Holcomb, A. L. Cohen, R. I. Gabitov, J. L. Hutter, Compositional and morphological features of aragonite precipitated experimentally from seawater and biogenically by corals. *Geochim. Cosmochim. Acta* **73**, 4166–4179 (2009).

63. D. Zoccola, E. Tambutté, F. Sénégas-Balas, J. F. Michiels, J. P. Failla, J. Jaubert, D. Allemand, Cloning of a calcium channel alpha1 subunit from the reef-building coral, Stylophora pistillata. *Gene* **227**, 157–167 (1999).

64. C. Brahmi, C. Kopp, I. Domart-coulon, J. Stolarski, A. Meibom, Skeletal growth dynamics linked to trace-element composition in the scleractinian coral *Pocillopora damicornis*. *Geochim. Cosmochim. Acta* **99**, 146–158 (2012).

65. J. Stolarski, Three − dimensional micro − and nanostructural characteristics of the scleractinian coral skeleton: A biocalcification proxy. *Acta Palaeontol. Pol.* **48**, 497–530 (2003).

66. A. Meibom, H. Yurimoto, J. P. Cuif, I. Domart-Coulon, F. Houlbreque, B. Constantz, Y. Dauphin, E. Tambutté, S. Tambutté, D. Allemand, J. Wooden, R. Dunbar, Vital effects in coral skeletal composition display strict three-dimensional control. *Geophys. Res. Lett.* **33**, 10.1029/2006GL025968 (2006).

67. A. Meibom, J.-P. Cuif, F. Houlbreque, S. Mostefaoui, Y. Dauphin, K. L. Meibom, R. Dunbar, Compositional variations at ultra-structure length scales in coral skeleton. *Geochim. Cosmochim. Acta* **72**, 1555–1569 (2008).

68. M. Park, Q. Li, N. Shcheynikov, W. Zeng, S. Muallem, NaBC1 is a ubiquitous electrogenic Na^{+}-coupled borate transporter essential for cellular boron homeostasis and cell growth and proliferation. *Mol. Cell* **16**, 331–341 (2004).

69. W. Guo, Seawater temperature and buffering capacity modulate coral calcifying pH. *Sci. Rep.* **9**, 1189 (2019).

70. E. M. Hemond, S. T. Kaluziak, S. V. Vollmer, The genetics of colony form and function in Caribbean *Acropora* corals. *BMC Genomics* **15**, 1133 (2014).

71. I. Taubner, F. Böhm, A. Eisenhauer, E. Tambutté, S. Tambutté, S. Moldzio, M. Bleich, An improved approach investigating epithelial ion transport in scleractinian corals. *Limnol. Oceanogr. Methods* **15**, 753–765 (2017).

72. S. Chen, A. C. Gagnon, J. F. Adkins, Carbonic anhydrase, coral calcification and a new model of stable isotope vital effects. *Geochim. Cosmochim. Acta* **236**, 179–197 (2018).

73. A. C. Gagnon, A. M. Gothmann, O. Branson, J. W. B. Rae, J. A. Stewart, Controls on boron isotopes in a cold-water coral and the cost of resilience to ocean acidification. *Earth Planet. Sci. Lett.* **554**, 116662 (2021).

74. T. Mass, A. J. Giuffre, C.-Y. Sun, C. A. Stifler, M. J. Frazier, M. Neder, N. Tamura, C. V. Stan, M. A. Marcus, P. U. P. A. Gilbert, Amorphous calcium carbonate particles form coral skeletons. *Proc. Natl. Acad. Sci. U.S.A.* **114**, 201707890 (2017).

75. P. Ganot, E. Tambutté, N. Caminiti-Segonds, G. Toullec, D. Allemand, S. Tambutté, Ubiquitous macropinocytosis in anthozoans. *eLife* **9**, e50022 (2020).

76. J. V. Büscher, A. U. Form, U. Riebesell, Interactive effects of ocean acidification and warming on growth, fitness and survival of the cold-water coral *Lophelia pertusa* under different food availabilities. *Front. Mar. Sci.* **4**, 101 (2017).

77. J. Fietzke, M. Frische, Experimental evaluation of elemental behavior during LA-ICP-MS: Influences of plasma conditions and limits of plasma robustness. *J. Anal. At. Spectrom* **31**, 234–244 (2016).

78. J. Fietzke, A. Heinemann, I. Taubner, F. Böhm, J. Erez, A. Eisenhauer, Boron isotope ratio determination in carbonates via LA-MC-ICP-MS using soda-lime glass standards as reference material. *J. Anal. At. Spectrom* **25**, 1953–1957 (2010).

79. M. Wall, F. Prada, J. Fietzke, E. Caroselli, Z. Dubinsky, L. Brizi, P. Fantazzini, S. Franzellitti, T. Mass, P. Montagna, G. Falini, S. Goffredo, Linking internal carbonate chemistry regulation and calcification in corals growing at a mediterranean CO_2 vent. *Front. Mar. Sci.* **6**, 699 (2019).

80. D. Mayk, J. Fietzke, E. Anagnostou, A. Paytan, LA-MC-ICP-MS study of boron isotopes in individual planktonic foraminifera: A novel approach to obtain seasonal variability patterns. *Chem. Geol.* **531**, 119351 (2020).

81. C. D. Standish, T. B. Chalk, T. L. Babila, J. A. Milton, M. R. Palmer, G. L. Foster, The effect of matrix interferences on *in situ* boron isotope analysis by laser ablation multi-collector inductively coupled plasma mass spectrometry. *Rapid Commun. Mass Spectrom.* **33**, 959–968 (2019).

82. A. Sadekov, N. S. Lloyd, S. Misra, J. Trotter, J. D'Olivo, M. McCulloch, Accurate and precise microscale measurements of boron isotope ratios in calcium carbonates using laser ablation multicollector-ICPMS. *J. Anal. At. Spectrom* **34**, 550–560 (2019).

83. D. Evans, A. Gerdes, D. Coenen, H. R. Marschall, W. Müller, Accurate correction for the matrix interference on laser ablation MC-ICPMS boron isotope measurements in $CaCO_3$ and silicate matrices. *J. Anal. At. Spectrom* **36**, 1607–1617 (2021).

84. F. J. Millero, T. B. Graham, F. Huang, H. Bustos-Serrano, D. Pierrot, Dissociation constants of carbonic acid in seawater as a function of salinity and temperature. *Mar. Chem.* **100**, 80–94 (2006).

85. K. Lee, T.-W. Kim, R. H. Byrne, F. J. Millero, R. A. Feely, Y.-M. Liu, The universal ratio of boron to chlorinity for the North Pacific and North Atlantic oceans. *Geochim. Cosmochim. Acta* **74**, 1801–1811 (2010).
